# Gene Expression during the Generation and Activation of Mouse Neutrophils: Implication of Novel Functional and Regulatory Pathways

**DOI:** 10.1371/journal.pone.0108553

**Published:** 2014-10-03

**Authors:** Jeffrey A. Ericson, Pierre Duffau, Kei Yasuda, Adriana Ortiz-Lopez, Katherine Rothamel, Ian R. Rifkin, Paul A. Monach, ImmGen Consortium

**Affiliations:** 1 Division of Immunology, Department of Microbiology and Immunology, Harvard Medical School, Boston, MA, United States of America; 2 Department of Medicine, Boston University School of Medicine, Boston, MA, United States of America; Wayne State University, United States of America

## Abstract

As part of the Immunological Genome Project (ImmGen), gene expression was determined in unstimulated (circulating) mouse neutrophils and three populations of neutrophils activated in vivo, with comparison among these populations and to other leukocytes. Activation conditions included serum-transfer arthritis (mediated by immune complexes), thioglycollate-induced peritonitis, and uric acid-induced peritonitis. Neutrophils expressed fewer genes than any other leukocyte population studied in ImmGen, and down-regulation of genes related to translation was particularly striking. However, genes with expression relatively specific to neutrophils were also identified, particularly three genes of unknown function: *Stfa2l1*, *Mrgpr2a* and *Mrgpr2b*. Comparison of genes up-regulated in activated neutrophils led to several novel findings: increased expression of genes related to synthesis and use of glutathione and of genes related to uptake and metabolism of modified lipoproteins, particularly in neutrophils elicited by thioglycollate; increased expression of genes for transcription factors in the Nr4a family, only in neutrophils elicited by serum-transfer arthritis; and increased expression of genes important in synthesis of prostaglandins and response to leukotrienes, particularly in neutrophils elicited by uric acid. Up-regulation of genes related to apoptosis, response to microbial products, *NFkB* family members and their regulators, and MHC class II expression was also seen, in agreement with previous studies. A regulatory model developed from the ImmGen data was used to infer regulatory genes involved in the changes in gene expression during neutrophil activation. Among 64, mostly novel, regulatory genes predicted to influence these changes in gene expression, *Irf5* was shown to be important for optimal secretion of IL-10, IP-10, MIP-1α, MIP-1β, and TNF-α by mouse neutrophils in vitro after stimulation through TLR9. This data-set and its analysis using the ImmGen regulatory model provide a basis for additional hypothesis-based research on the importance of changes in gene expression in neutrophils in different conditions.

## Introduction

The Immunological Genome Project (ImmGen) is a consortium of immunologists and computational biologists who aim to produce a comprehensive description of gene expression and a model of its regulation in the immune system of the mouse [1]–[11]. In this context, we analyzed gene expression in neutrophils, in order to determine gene expression patterns that distinguish neutrophils from other leukocytes, compare expression patterns among neutrophils activated by different stimuli in vivo, and infer regulators of gene expression during neutrophil activation using the ImmGen regulatory model.

Neutrophils are highly differentiated cells of the myeloid lineage and are produced in large numbers in the bone marrow. They are then released into the circulation, from which they extravasate in response to a variety of inflammatory stimuli. Neutrophils are specialized for defense against bacterial infection and are essential for host survival in a normal environment. However, “acute” neutrophilic inflammation is also characteristic of diverse non-infectious disease states such as inflammatory arthritis, neutrophilic dermatoses, and vascultis.

Unstimulated neutrophils are short-lived, and many of the best-known functions of activated neutrophils involve pre-formed mediators. However, over the past 25 years it has become clear that activated neutrophils have prolonged survival, that they undergo prominent changes in gene expression, and that they synthesize and secrete proteins [12]–[15], indicating that studies of gene expression are biologically relevant. Gene expression profiling of neutrophils has been reported in multiple studies, mostly for human cells, sometimes ex vivo comparing disease states [16]–[19] but more often in vitro after stimulation with lipopolysaccharide, GM-CSF, or bacteria [19]–[24]. In all of these studies, numerous changes in gene expression were seen with neutrophil activation. Two findings noted in multiple studies have been up-regulation of anti-apoptotic genes [17], [18], [23], [24] and genes for pro-inflammatory cytokines and chemokines [17], [18], [20], [21], [24]. Some authors have focused on other changes, such as in genes for transcription factors [22] or related to antigen presentation [19], and these papers have also reported differences among different stimuli in vitro [19], [22]. We are aware of only one study of gene expression in mouse neutrophils, in which neutrophils activated in vivo by thioglycollate-induced peritonitis were found to express many genes previously thought to be specific to macrophages [25]. Mouse neutrophils activated in vivo by different stimuli have not been compared to each other, nor to non-activated neutrophils.

The importance of particular regulators of gene expression has been established most conclusively for the differentiation of neutrophils; for example, PU.1, CEBP/α, CEBP/ε, and Gfi-1 are essential for normal granulopoiesis [26]–[29]. During neutrophil activation, studied using human cells in vitro, evidence for involvement of STAT proteins, NFκB isoforms (specifically the canonical pathway involving NFκB1/p50 and RelA), and CEBP/α has been obtained [26], [30].

In the current study, we obtained gene expression profiles from unstimulated mouse neutrophils (bone marrow and blood) and three disease states that involve extravasation and activation, in order to identify genes that distinguish neutrophils from other leukocytes, to identify changes in gene expression that are shared among activated states, and to identify changes characteristic of a particular stimulus. Uric acid (UA) crystals elicit inflammation in the peritoneal cavity–a model for the human arthritic disease gout–and initiate pro-inflammatory signals in leukocytes through the NLRP3 inflammasome [31]. Thioglycollate broth (TG) elicits neutrophilic and then macrophage inflammation in the peritoneal cavity; this technique has been used for many years to study neutrophils and especially macrophages, but no specific human disease is modeled. The mechanism is undefined, but since yeast extract is a component of the broth, signaling through multiple innate-immune receptors is likely. Autoantibodies to glucose-6-phosphate isomerase produce inflammatory arthritis with similarities to the human disease rheumatoid arthritis. Neutrophils infiltrate the synovial fluid (SF), through deposition of immune complexes in the joint [32], [33]. In all of these models, neutrophils circulating in the blood are the precursors of the cells accumulating in the inflamed sites and are an appropriate standard for comparison. The fact that this project was part of ImmGen allowed an additional and novel analysis: we used the ImmGen regulatory model [10] to infer the importance of many transcription factors in neutrophil activation.

## Methods

### Ethics Statement

All experiments using mice were conducted under protocols approved by the HMA Standing Committee on Animals of Harvard Medical School or the Institutional Animal Care and Use Committee of the Boston University Medical Campus.

### Mice

For experiments involving gene expression profiling, male C57BL/6 mice were purchased from the Jackson Laboratory at five weeks of age and maintained at Harvard Medical School for one week before use in experiments.

For experiments using neutrophils in vitro, C57BL/6 wild-type mice were purchased from the Jackson Laboratory. *Irf5*−/− mice (backcrossed 8 generations to C57BL/6) were provided by Dr. T. Taniguchi (University of Tokyo, Tokyo, Japan) and Dr. T. Mak (University of Toronto, Toronto, Canada) [34] and then backcrossed a further 7 generations to C57BL/6 mice from the Jackson Laboratory. Mice were maintained at the Boston University School of Medicine Laboratory Animal Sciences Center and used under IACUC-approved protocol 14794.

### Inflammatory Stimuli and Collection of Cells

Arthritis was induced using serum from K/BxN mice, 0.15 ml intraperitoneally (i.p.) on day 0 and day 2. Synovial fluid was collected on day 7 by puncture of the medial or lateral ankle with a 21-gauge needle, recovery of the fluid with a micropipet, and immediate dilution in cold DMEM (without Phenol Red) containing 5% FBS, 0.1% sodium azide (DMEM/FBS/azide), and 20 mM EDTA. Peritonitis was induced by i.p. injection of 1 ml autoclaved 3% thioglycollate FTG medium (Sigma), or 0.1 ml of 10% uric acid (Sigma, non-crystalline) in 0.8% NaCl that had been sonicated and stored at RT overnight to allow crystals to form [35]. Peritoneal exudate cells were recovered 18 hr later by lavage with 9 ml cold DMEM/FBS/azide. Blood was collected by cardiac puncture and immediately diluted into cold DMEM/FBS/azide also containing 20 mM EDTA. Bone marrow cells from femurs were extruded directly into cold DMEM/FBS/azide.

### Purification of Neutrophils, Flow Cytometry

In most cases, samples from two mice were pooled before purification of neutrophils for gene expression studies. The standard ImmGen protocol for staining and fluorescence activated cell sorting (FACS) was used (www.immgen.org/Protocols/ImmGen%20Cell%20prep%20and%20sorting%20SOP.pdf), including a maximum of 2 hours between mouse sacrifice and staining. RBC were removed by hypotonic lysis with ACK medium for 3 min on ice for most samples. Removal of RBC from blood samples required two treatments of 5–10 min each. The remaining cells were stained with PE-conjugated anti-CD11b (clone M1/70, eBioscience) and APC-Cy7-conjugated anti-Ly6G (clone 1A8, BD Pharmingen) in DMEM/FBS/azide for 10 min, and neutrophils were recovered by FACS (FACS Aria, Becton Dickinson) based on high side-scatter, bright staining for Ly6G and CD11b, and exclusion of doublets. Two cycles of FACS were performed, and purity of the sorted cells was at least 99% after the second sort. Fifty thousand cells were sorted directly into TRIzol Reagent (Invitrogen) for recovery of RNA during the second sort. Common myeloid precursors (CMP) were sorted as Lin^−^IL7R-Sca1^−^cKit^+^FcgR^l^°CD34^+^ cells, and granulocyte/monocyte precursors (GMP) as Lin^−^IL7R-Sca1^−^cKit^+^FcgR^hi^CD34^+^ cells. For purification of other leukocyte populations, see www.immgen.org. For purification of splenic leukocyte populations for gene expression analysis by RNA-Seq, see www.immgen.org/Protocols/11cells.pdf.

For purification of neutrophils for subsequent stimulation in vitro, see below.

### RNA Processing, Microarrays, and Data Processing

RNA purity was determined using an Agilent 2100 bioanalyzer, and all samples had RNA Integrity (RIN) scores greater than 7 (on a scale of 0–10), the standard for inclusion in ImmGen. Per standard ImmGen protocol (www.immgen.org/Protocols/Total%20RNA%20Extraction%20with%20Trizol.pdf), RNA was amplified and hybridized to the Affymetrix MoGene 1.0 ST array with the GeneChip Whole Transcript (WT) Sense Target Labeling Assay per the manufacturer’s instructions. Raw data were normalized using the GenePattern module ExpressionFileCreator and its robust multichip average algorithm. Isolation of polyA+ RNA, RNA-Seq, and analysis of RNA-Seq data were performed as described in www.immgen.com/Protocols/11cells.pdf.

Gene Expression Omnibus accession number: GSE15907.

### Filtering of Genes to be Analyzed

For comparison of neutrophils to non-neutrophil leukocytes, data from all probes on the array were used. Analyses comparing neutrophil populations to each other or inferring regulatory genes were limited to genes with mean expression >120 after normalization in at least one neutrophil population, since this level of expression on the 1.0 ST array has been associated with a 95% chance of protein expression and is being routinely used as the cut-off value in ImmGen studies [36]. Significant variation across neutrophil populations (ANOVA P<0.01), fold-difference ≥2 in at least one pair-wise comparison of populations, and acceptable variation within replicates (within-group coefficient of variation (CV) <0.5 across neutrophil populations) were also used as filters for these analyses.

### Analysis of Gene Ontology Categories and Functional Pathways

The distribution of genes into Gene Ontology (GO) categories and Kyoto Encyclopedia of Genes and Genomes (KEGG) pathways was analyzed using DAVID (http://david.abcc.ncifcrf.gov/) and its default Benjamini-Hochberg adjustment for multiple comparisons, with adjusted Q<0.05 regarded as significant. The Functional Annotation Clustering tool in DAVID was used to identify redundant GO categories and KEGG pathways.

Lists of genes analyzed using DAVID included genes over-expressed or under-expressed in all neutrophil populations compared to all non-neutrophil populations; genes up- or down-regulated in SF, TG, or UA neutrophils relative to blood neutrophils (2-fold or 1.5-fold); genes up- or down-regulated at least 2-fold in SF, TG, or UA compared to all other neutrophil populations; and genes implicated in a shared regulatory network (see below). The numbers of genes in different GO categories or KEGG pathways that were up- or down-regulated in these three activating conditions were analyzed by Fisher’s exact test or chi-square test in pairwise comparisons.

Categories and pathways of interest were studied in more detail. Among genes down-regulated in neutrophils compared to non-neutrophils, expression of all genes in the significantly enriched GO categories (www.geneontology.org) was analyzed. For comparison of activated neutrophil populations, compilation of significant GO terms and KEGG pathways was supplemented by manual examination of gene lists and refinement of pathways after consultation of the NCBI Gene annotations (ncbi.nlm.nih.gov/gene) and the literature via PubMed. For analysis of expression data for genes on the most of the resulting lists, filters for expression (>120), significance (ANOVA Q<0.05 after adjustment for multiple comparisons), and fold change (>2 in a pairwise comparison, in most cases) were retained, but the filter for CV was removed due to the small numbers of genes being analyzed simultaneously and incorporation of such variation into analysis by ANOVA. For the list of genes in a pathway implicated only after analysis of likely regulatory genes (see below), the fold-change criterion was relaxed (>1.5) and the ANOVA criterion removed, with genes not meeting the more strict criteria being noted. Graphics were created using the Pathway Designer function of Ingenuity Systems (www.ingenuity.com).

### Visualization of Differences in Gene Expression

Global gene expression patterns in leukocyte populations were compared by principal components analysis (PCA) using the ‘Population PCA’ tool (http://cbdm.hms.harvard.edu/LabMembersPges/SD.html). Heat maps were produced using GenePattern module HeatMapImage. For comparison of expression among neutrophil populations (blood, SF, UA, and TG), expression was log-transformed and mean-centered across the 4 populations for each gene. The gradient was set to indicate an 8-fold difference between lowest (dark blue) and highest (dark red) expression, so as to allow visualization of 2-fold differences and comparison among genes; for a few genes, the differences were larger than 8-fold and are not fully appreciable.

### Analysis Using the ImmGen Regulatory Model

Starting with the 1283 genes that had passed initial filters for expression level and variation between and within groups as above, expression data from individual replicates of neutrophils purified from blood, SF, TG or UA were used to place genes into clusters using ExpressCluster (http://cbdm.hms.harvard.edu/LabMembersPges/SD/downloads/ExpressCluster_v1.3.pdf): K-means clustering with k = 32 clusters that converged after 13 iterations, using Euclidean distance as the distance metric with mean-centered signal transformation. Correlation coefficients were calculated for each cluster. Clusters showing similar patterns but differing in magnitude were merged for subsequent analyses (resulting in 25 clusters), and re-calculation of correlation coefficients confirmed that such merging was appropriate, since coefficients dropped little if at all (maximum drop 0.03). To assess the statistical significance of the clustering process, normalized expression values for all genes were randomized for each sample, and that simulated data-set was analyzed by ExpressCluster and correlation coefficients calculated in the same way.

In the ImmGen regulatory model, each gene is assigned to one coarse (n = 81) and one fine (n = 334) module based on correlated expression across all populations; each module is associated with multiple regulators, with associations assigned weights based on the beta-coefficients from a multiple regression equation unique to each module [10]. Thus, each target gene in a dataset generates multiple regulator-target pairs, and weights can be compared only within modules, not between them. Some regulators are associated with few modules, some with many, so the maximum number of regulator-target pairs is highly variable among regulators. Since there is no objectively “best” way to infer regulator importance using this framework, we performed two analyses in parallel: i) restricting the analysis to coarse modules with significantly increased numbers of genes, then compiling the regulators of those modules; and ii) for each regulator in light of its assignment to coarse modules, comparing the number of regulator-target pairs generated by a list of genes to the maximum number of regulator-target pairs in the model.

Distribution of genes into the 81 coarse modules was compared to a random distribution generated by simulation using custom PERL script that measured total bin counts after 10,000 sets of random distributions of X numbers into 81 bins with different sizes determined by the number of target genes in each of the coarse modules, where X is the number of unique genes in a particular cluster group that was a target gene member of one of the coarse modules. The 25 clusters were pooled into 3 groups (11 clusters of up-regulated genes, 9 clusters of down-regulated genes, and 5 with more complex patterns) in order to obtain acceptable statistical power. Q<0.05 after adjustment for the false discovery rate [37] was regarded as significant. Regulators associated with these modules were identified.

Analyses for enrichment of regulator-target pairs was also performed using these 3 pools of clusters. Over-representation of regulator-target pairs was determined by chi-square test with adjustment for the false discovery rate, with Q<0.01 chosen as the cut-off value in order to enrich for the most highly over-represented regulators [37]. The list of regulators chosen for further analysis included those that were over-represented in one of the 3 pools of clusters and also had been associated with a significantly enriched coarse module as above. The genes in each of the 25 individual clusters were then analyzed similarly for enrichment of regulator-target pairs, to create a matrix of P-values for each regulator with each cluster of expressed genes. This matrix was subjected to hierarchical clustering of both rows (regulators) and columns (clusters) to identify related regulators and related gene clusters. The HeatMapImage module in GenePattern was used for visualization.

### Neutrophil Stimulation in vitro

Neutrophils were first enriched from bone marrow on a 62.5% Percoll column [38], then stained with PE-conjugated anti-CD11b (clone M1/70, eBioscience), PerCP-Cy5.5-conjugated anti-Gr1 (i.e., anti-Ly6G and/or Ly6C, clone RB6-8C5, BD Pharmingen), and APC-conjugated anti-F4/80 (clone BM8, BioLegend). In one experiment, cells were stained with PE-conjugated anti-CD11b and FITC-conjugated anti-Ly6G (clone 1A8, BioLegend). Purified neutrophils (CD11b^+^Gr1^hi^F4/80^−^ or CD11b^+^Ly6G^hi^) were sorted by FACS on a MoFlo instrument (Beckman Coulter). The neutrophil population was >98% pure as assessed by Wright-Giemsa stain of cytospun samples.

Neutrophils were resuspended in RPMI-1640 medium supplemented with 10% fetal bovine serum, 2 mM L-glutamine, 100 U/ml penicillin and 100 µg/ml streptomycin (complete medium) and seeded at 3×10^5^ cells/well in 96-well round-bottom plates. They were then incubated with the following TLR ligands for 16 hours: the TLR2 ligand Pam3Cys-Ser-Lys4 (Pam3Cys) (100 ng/ml), the TLR3 ligand poly(deoxyinosinic-deoxycytidylic acid) (poly(I:C)) (10 ug/ml), the TLR4 ligand LPS (100 ng/ml), and the TLR9 ligand CpG-B (oligodeoxynucleotide (ODN) 1826) (1 ug/ml) (all from InvivoGen). Following incubation, the supernatants were collected and concentrations of selected cytokines and chemokines (IL-1β, IL-10, IP-10, KC, G-CSF, MIP-1α, MIP-1β, MIP-2, and TNF-α) in the supernatants were measured by Luminex (National Mouse Metabolic Phenotyping Center at the University of Massachusetts Medical Center, using reagents from Millipore).

## Results and Discussion

**Figure 1:**
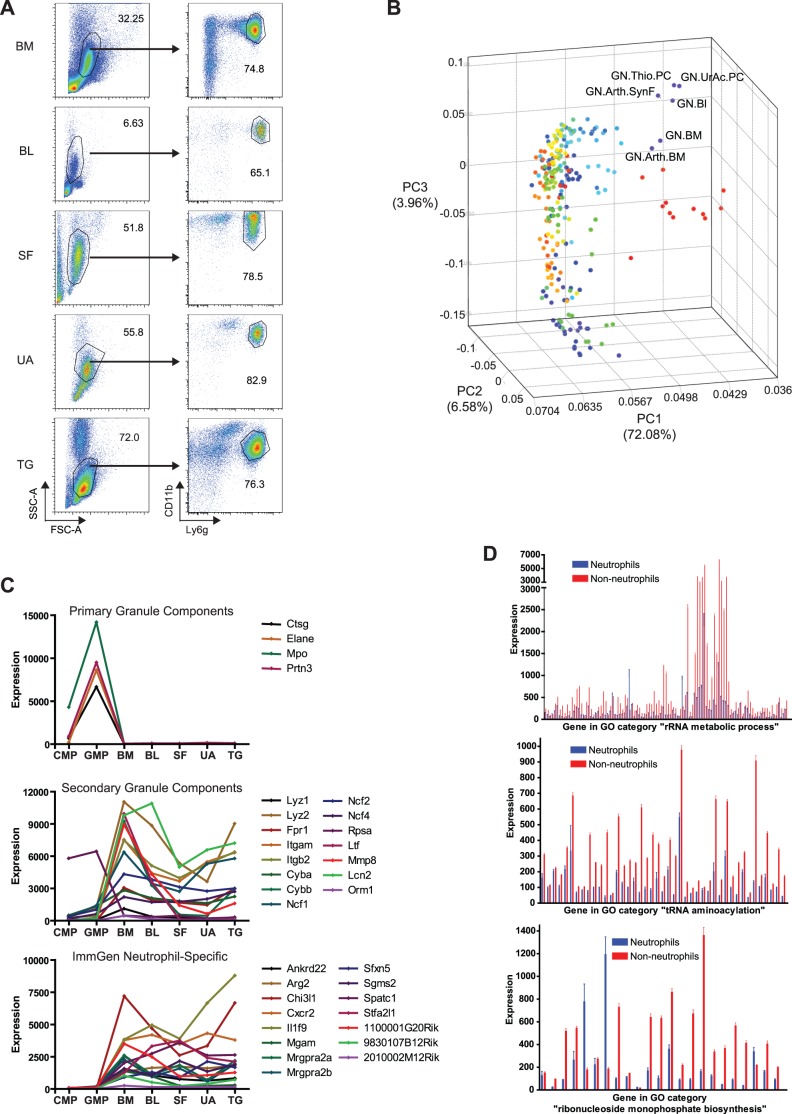
Isolation of neutrophils and characterization of gene expression patterns. **A.** Neutrophils were isolated from bone marrow (BM) and blood (BL) of untreated mice, from the peritoneal cavity of mice administered thioglycollate (TG) or uric acid (UA) intraperitoneally, and from the synovial fluid (SF) of mice with autoantibody-induced arthritis, on the basis of scatter patterns (which differed among conditions, left panels) and staining for CD11b and Ly6G (right panels). The population in the upper left corner of the TG plot did not express CD11b or Ly6G. **B.** Comparison of global gene expression patterns in neutrophils (labeled) to all of the other populations in ImmGen, using axes determined by principal components analysis (PCA). Populations in red on the right side of the diagram represent stromal cell populations; other colors represent various lymphoid and myeloid populations. To convert ImmGen nomenclature to the abbreviations used in this paper: Thio.PC = TG; UrAc.PC = UA; Arth.SynF = SF; GN.Bl = BL; GN.BM = BM = bone-marrow neutrophils from normal mice; Arth.BM = bone-marrow neutrophils from arthritic mice, note similarity to GN.BM. **C.** Expression of genes for components of neutrophil primary granules (top), secondary granules (middle), and 15 genes showing greater expression in neutrophils than non-neutrophils in ImmGen [mean expression among 5 neutrophil populations (BM, BL, SF, UA, and TG) being greater than 4 times the maximum expression among 198 non-neutrophil populations](bottom), during neutrophil development and activation. CMP = common myeloid precursor; GMP = granulocyte/monocyte precursor. Note that expression patterns in the “neutrophil-specific” genes as identified in this study resembled those of secondary but not primary granule components. **D.** Expression of groups of genes related to translation (per Gene Ontology = GO) in neutrophils (blue) and other leukocytes (red). Each bar represents mean expression among 5 neutrophil or 198 non-neutrophil populations, and error bars show standard errors.

Neutrophils were purified by FACS, on the basis of forward-scatter/side-scatter pattern and staining for Ly6G and CD11b [39], from bone marrow, blood, and three inflammatory conditions: SF 7 days after induction of arthritis using autoantibodies, peritonitis 18 hours after injection of TG, and peritonitis 18 hours after injection of UA (**Fig. 1A**). Cell purification was performed according to ImmGen standard operating protocols, from 5-week-old C57BL/6J male mice. Microarray gene expression profiles were generated on triplicate samples using ImmGen standard pipelines for data generation, processing, and quality control.

### Gene expression in neutrophil populations with comparison to other leukocytes

Based on analysis of global gene expression patterns by PCA, the 5 neutrophil populations clustered distinctly from all other leukocyte populations in ImmGen (**Fig. 1B**). This unique expression pattern was driven both by over-expression and under-expression of genes in neutrophils compared to the other 198 leukocyte populations: e.g., mean expression of 457 probes was at least 4-fold higher in neutrophils than non-neutrophils, and mean expression of 1179 probes was at least 4-fold lower. The total number of probes with absolute expression >120 (used as a cut-off because it is highly predictive of translation into detectable protein) [36] was lower in neutrophils (range 9166–9437) than in other leukocytes (mean 10198, range 9806–11342, P<0.0001 comparing neutrophils to any other type of leukocyte).

Genes with expression most specific to neutrophils were identified in two ways: i) genes reliably expressed (absolute expression >120) in all 5 neutrophil populations but in none of the 198 non-neutrophil populations, and ii) minimum expression among the 5 neutrophil populations at least 2-fold higher than the maximum expression in any other leukocyte. Thirteen genes met the first criterion, and 23 met the second, with 5 meeting both criteria (**Table 1**). These 31 genes were not enriched in any Gene Ontology (GO) term, and no functional theme was evident by inspection. Data obtained from splenic leukocyte populations using RNA-Seq, a different method to quantify mRNA, confirmed neutrophil-specific expression of these genes (**Table S1)**. Eosinophils have not yet been profiled in ImmGen, but published data using the same microarray platform [40] indicate that 10 of these 31 genes, including only 3 of the 13 genes expressed in all neutrophil populations but no other leukocytes in ImmGen, are definitely expressed in eosinophils (**Table 1**).

**Table 1:** Genes with expression most specific to neutrophils in the ImmGen database.

Gene Symbol	BM	BL	SF	UA	TG	Median (Range) NF	Median (Range) Non-NF	Non-NF>120*	Mean Eos.†	Hume Sig.‡
1100001G20Rik	3506	2490	980	1064	1279	1279 (980–3506)	92 (64–376)	12	119	Y
2010002M12Rik	262	143	152	164	146	152 (143–262)	16 (11–38)	0	13	Y
9830107B12Rik	1028	518	230	396	724	518 (230–1028)	20 (14–63)	0	1321	0
Ankrd22	2569	1065	766	641	828	828 (641–2569)	41 (27–153)	2	58	Y
Arg2	1345	1641	1732	1606	1810	1641 (1345–1810)	40 (27–316)	5	941	Y
Ceacam10	1489	542	247	253	168	253 (168–1489)	30 (22–116)	0	38	Y
Chi3l1	7201	4700	2612	3346	6669	4700 (2612–7201)	49 (35–549)	9	82	Y
Clec5a	3264	1979	1966	1908	3421	1980 (1908–3421)	24 (15–797)	33	32	0
Csf3r	3146	5917	3274	6041	5337	5337 (3146–6041)	37 (23–1068)	40	93	Y
Cxcr2	3777	4194	3476	4324	3799	3799 (3476–4324)	15 (12–466)	5	711	Y
Dhrs9	1522	1795	1113	1763	1594	1594 (1113–1795)	28 (19–420)	8	692	0
Fam123a	283	259	310	207	122	259 (122–310)	49 (32–93)	0	69	0
Gm9949	210	314	154	400	231	231 (154–400)	54 (36–106)	0	77	0
Grina	5809	7197	7489	8429	7112	7197 (5809–8429)	533 (184–2205)	198	2697	0
Il1f9	3836	4955	3896	6662	8800	4955 (3836–8800)	19 (14–262)	3	109	Y
Kctd11	126	140	121	137	180	137 (121–180)	58 (42–109)	0	117	0
Mgam	1389	982	256	247	294	294 (247–1389)	32 (23–112)	0	101	Y
Mir29c	218	186	155	614	537	218 (155–614)	28 (13–113)	0	230	0
Mrgpra2a	2605	1103	1856	697	2200	1856 (697–2605)	41 (27–213)	4	123	Y
Mrgpra2b	2279	950	1589	568	1949	1589 (568–2279)	41 (29–194)	3	121	Y
Ppp1r3d	788	1018	719	1091	645	788 (645–1091)	133 (93–280)	143	150	Y
Prrg2	137	181	155	152	155	155 (137–181)	63 (46–108)	0	82	0
Rlf	1213	1083	1031	1268	1315	1213 (1031–1315)	344 (80–513)	195	523	0
Rnf11	1139	1193	1390	1036	931	1139 (931–1390)	248 (114–451)	197	356	0
S100a7a	148	154	128	235	199	154 (128–235)	84 (67–116)	0	100	0
Sfxn5	915	1308	885	2129	1671	1308 (885–2129)	75 (47–269)	19	126	Y
Sgms2	1580	1060	2172	1352	1890	1580 (1060–2172)	40 (26–374)	8	2211	0
Slc22a20	138	139	149	137	136	138 (136–149)	77 (50–107)	0	128	0
Slfn4	5074	4516	3032	9288	11010	5074 (3032–11010)	42 (31–1455)	11	79	Y
Spatc1	2020	2748	3598	2597	2640	2640 (2020–3598)	32 (22–119)	0	626	0
Stfa2l1	1270	3317	3750	2409	2120	2409 (1270–3750)	10 (8–107)	0	16	Y

Numbers indicate gene expression levels. BM = bone-marrow neutrophils; BL = blood neutrophils; SF = synovial fluid neutrophils; UA = uric acid-induced peritoneal neutrophils; TG = thioglycollate-induced peritoneal neutrophils; NF = neutrophils; Non-NF = all non-neutrophil leukocyte populations profiled in ImmGen.

*The number of non-neutrophils populations (of 198 total) in which expression was greater than 120.

† Published expression in eosinophils using the same microarray platform [40].

‡ Included in a published gene-expression signature for neutrophils [47].

Two of the 31 genes (*Csf3r* and *Cxcr2*) are well-known to be important in neutrophil biology and to be relatively but not completely specific to neutrophils. Four other genes (*Chi3l1, Clec5a, Mgam*, and *Sgms2*) have been studied in neutrophils but also in other leukocytes [41]–[46]. The remaining 25 genes have not been specifically studied in neutrophils; expression of 12 of them has been reported to be relatively specific to neutrophils compared to other leukocytes in an analysis of the BioGPS database [47], but the other 13 did not appear in that signature (see **Table 1**).

Considering specificity beyond the hematopoietic system, 7 of the 12 previously described and 11 of the 13 novel genes have been reported to be expressed in at least one non-hematopoietic cell type. Expression data were surveyed via the BioGPS website (biogps.org) for the remaining 7 genes for which there was no literature on expression, leading to the conclusion that *Stfa2l1* and *Mrgpr2a* and *b* (genes of unknown function) are particularly likely to be specific to neutrophils. In our data-set, these genes were not expressed in myeloid precursors, were highly expressed in mature bone-marrow neutrophils, and continued to be expressed during neutrophil circulation and activation. *Stfa2l1* was not expressed significantly in any non-neutrophil population, and the minimum expression in neutrophils was 12-fold higher than the maximum expression in non-neutrophil leukocytes (**Table 1**). In the MOE430 Gene Atlas data-set (inspected on biogps.org), *Stfa2l1* was expressed in mature granulocytes and bone marrow, but otherwise only in umbilical cord at a low level. *Mrgpra2a* and *b* were expressed at low levels in 3–4 non-neutrophil populations in our data-set, with minimum neutrophil expression 3-fold higher than maximum non-neutrophil expression; in the MOE430 data-set, expression was high in mature granulocytes and bone marrow, but otherwise only seen in the dorsal root ganglia.

Despite the unclear functional significance, these results are consistent with the literature. As above, 16/31 genes (including *Stfa2l1* and *Mrgpr2a* and *b*) were among the 206 genes assigned to neutrophil/granulocyte-oriented clusters in analyses of the BioGPS dataset [47]. In turn, 155 of those 206 genes could be assessed in our data-set; the great majority were highly expressed in neutrophils, particularly in unstimulated cells, but some declined in activated cells and many were also expressed in other leukocytes (**File S1**).

In contrast, genes encoding components of neutrophil granules had two different patterns of expression, consistent with the literature [48]–[50]: expression of primary (azurophilic) granule components was virtually limited to granulopoiesis (granulocyte-monocyte precursor, GMP), and expression of components of secondary (specific), tertiary, or secretory granules peaked at the mature stage in the bone marrow and declined among circulating and activated cells (**Fig. 1C**). In agreement with these findings, published analyses of BioGPS and other databases have also incorporated a few genes for secondary/tertiary granule proteins (*Fpr1, Lcn2, Ltf, Orm1, Mmp8*), but no genes for primary granule proteins, into granulocyte signatures [47], [51]. Thus, the canonical proteins of neutrophils do not serve as an ideal genetic signature for mature neutrophils, which has implications regarding strategies for identifying evidence of neutrophil infiltration or contamination in studies of complex tissues. The highly neutrophil-specific genes we have identified, such as *Stfa2l1* and *Mrgpra2a* and *b*, are good candidates for development of Cre-expressing mice with greater specificity for neutrophils than the best currently-available model based on human *MRP8* [52] (equivalent to mouse *S100a8*, which showed good specificity in our studies as well but did not meet our strict criteria, see immgen.org). The genes of unknown function in **Table 1** are also good candidates for study related to unique neutrophil actions such as NETosis.

The genes most specifically under-expressed in neutrophils were identified in an analogous way as with over-expressed genes. Sixty-five probes had minimum expression in non-neutrophils more than 2-fold greater than maximum expression in neutrophils, 98 probes were expressed in all 198 non-neutrophil populations but in none of the 5 neutrophil populations, and 17 probes met both criteria, leaving 146 probes associated with 120 genes. These genes were significantly enriched in GO categories related to translation, e.g., rRNA metabolic process, rNMP biosynthesis, tRNA aminoacylation, nucleocytoplasmic transport (all Q<0.05 after Benjamini-Hochberg adjustment), and related/redundant categories. Almost all of the remaining genes (i.e., those that did not meet the strict criteria for being specifically under-expressed) in the first 3 of these categories were also expressed at lower levels in neutrophils than non-neutrophils (**Fig. 1D**). These results are consistent with the previously-described scarcity of ribosomes in neutrophils [53] and probably reflect a conservation of energy for other processes during a short lifespan. Although limited availability of ribosomes and/or tRNAs is probably the reason that gene expression in neutrophils does not always correlate with protein production [12], [20], it remains unclear whether there is a mechanism for prioritization for translation above and beyond merely the relative abundance of different mRNAs [12].

### Comparison of neutrophils activated by different stimuli

As described in more detail in **Text S1** and **Figure S1**, changes in gene expression in SF, TG, and UA neutrophils were compared using plots comparing fold-changes relative to blood neutrophils, Venn diagrams, and statistical analysis of distribution into GO categories. To summarize, the majority of differences were quantitative rather than qualitative. In particular, correlation was high (r = 0.79) between TG and UA, but changes in TG were of greater magnitude. The lowest correlation was between SF and UA (r = 0.55), and very few changes in gene expression were seen in both SF and UA but not TG. Down-regulation of individual genes was more likely to be shared among all 3 conditions than was up-regulation.

Because these analyses had low power to detect changes in small numbers of related genes, we also identified every gene with expression in TG, SF, or UA that was at least 2-fold higher than in either of the other conditions and in blood. Seventy-nine genes were relatively specific for TG, 49 for SF, and 13 for UA (**Table S2**). Inspection of these lists revealed several groups of genes with shared functions. TG neutrophils up-regulated NFκB subunits and regulators, enzymes involved in gluthathione metabolism and other antioxidants, and signaling molecules in pathways for responding to microbial products. SF neutrophils up-regulated MHC class II genes, the *C1q* component of complement, all 3 members of the Nr4a nuclear hormone receptor subgroup (*Nr4a1*, *2*, and *3*), and molecules related to the uptake and metabolism of lipoproteins. UA neutrophils up-regulated two receptors for leukotrienes (*Cysltr1* and *Ltb4r1*). Genes specifically down-regulated in TG (n = 3), SF (n = 42), or UA (n = 9) did not contain any shared functions that were obvious on inspection, nor by analysis using DAVID.

We proceeded to analyze and interpret the functions of genes up-regulated or down-regulated in activated neutrophils, whether shared among activating conditions or relatively specific to one condition.

### Functions of genes up-regulated in activated neutrophils

Most of the GO biological processes in which genes up-regulated in activated neutrophils were enriched were very broad and not surprising: apoptosis, regulation of apoptosis, immune system development, cellular ion homeostasis, inflammatory response, regulation of leukocyte activation, regulation of cytokine production, response to oxidative stress, positive regulation of catalytic activity, phosphorus metabolic process, regulation of small GTPase mediated signal transduction, protein homooligomerization, and negative regulation of cell proliferation. We focused further analysis on the 10 significant GO terms with 50 or fewer genes. These more-specific terms still often shared genes, which allowed functions of interest to be summarized as: i) regulation of apoptosis, ii) pro-inflammatory signaling through NFκB including pathways for responding to microbial products, iii) glutathione metabolism, and iv) antigen processing and presentation. Analysis for enrichment in KEGG pathways corroborated the first 3 of these functions and also indicated that genes for lysosome components, not surprisingly, were significantly up-regulated in all three activated populations. Inspection of genes specifically up-regulated in one activating condition (**Table S2**) had also indicated that the latter 3 of these functions were of interest, as were metabolism of lipoproteins, Nr4a-family nuclear receptors, and receptors for leukotrienes.

#### Uptake and metabolism of modified lipoprotein

**Figure 2:**
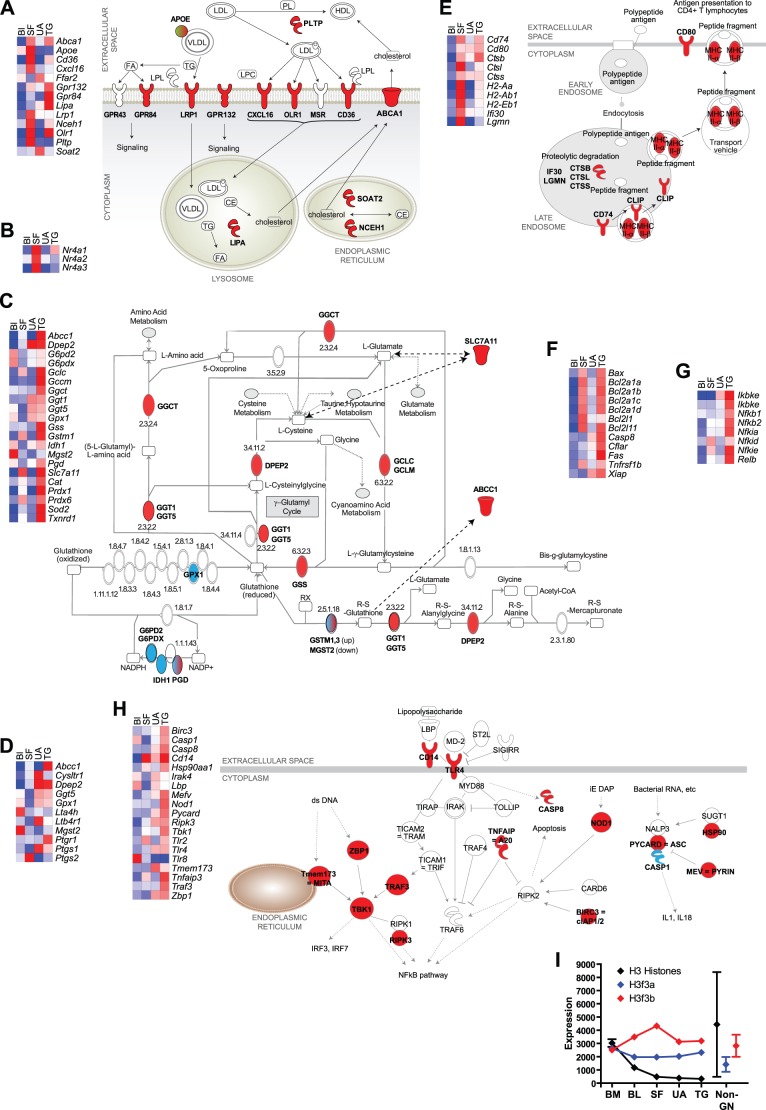
Biological processes showing up-regulation or down-regulation of genes in activated neutrophils. (**A–H**). Heat maps show mean expression in neutrophils from blood (BL), synovial fluid (SF), or peritonitis induced by uric acid (UA) or thioglycollate (TG). Mean expression across all four conditions was placed at the center of the gradient (white) for each gene. Red indicates increased expression, and blue indicates decreased expression. The full color gradient for each gene represents an 8-fold difference in expression. Lists of genes of interest were compiled using the KEGG and Ingenuity databases as well as literature reviews; only genes showing at least 2-fold differences in expression comparing conditions and with Q<0.05 by ANOVA are shown. In the pathway diagrams, up-regulated genes are shown in red, and down-regulated genes are shown in green. **A.** Uptake and metabolism of lipoproteins. **B.** Nr4a-family transcription factors. **C.** Glutathione metabolism. **D.** Synthesis of and response to leukotrienes and prostaglandins. **E.** Antigen processing and presentation via MHC class II. **F.** Genes related to apoptosis. **G.** NFκB subunits and proximal regulators of NFκB. **H.** Genes related to signaling by innate immune receptors for microbial products. **I.** Expression of H3 histone genes (*Hist1h3a, b, c, d, e, g, h, I,* and *Hist2h3b* and *3c1*) in neutrophil populations. Mean ± SD of these 10 genes (black) declined after release from bone marrow (BM) to blood (BL) and further after activation (SF, UA, TG). Mean ± SD among 198 non-neutrophil populations is shown for comparison. Although it is not apparent from this plot, the lowest expression among non-neutrophils exceeded the highest expression in UA or TG neutrophils. Expression of genes for the “replacement” H3 histones, shown in red and blue, was maintained after neutrophil maturation and activation, at levels similar to non-neutrophils.

Up-regulation of multiple endocytic receptors for VLDL (*Lrp1*) and oxidized LDL (*Cxcl16*, *Olr1*, *Cd36*) was most prominent in SF neutrophils, whereas induction of lysosomal lipase (*Lipa*) and signaling receptors for lysophosphatidylcholine (*Gpr132*) and free fatty acids (*Gpr84*) was most characteristic of TG neutrophils (**Fig. 2A**). Uptake of modified lipoproteins, breakdown of triglycerides and cholesterol esters, and export of cholesterol are all well-described in macrophages, and dysfunction of this system is important in foam cell formation in atherosclerosis [54], [55]. Similar mechanisms are not known to operate in neutrophils. No previous studies have commented on up-regulation of genes related to lipoprotein metabolism, but corroboration of this finding at the level of gene expression is provided by review of data from human neutrophils stimulated in vitro: transcripts for *CD36, CXCL16, GPR132, LRP1, OLR1,* and additionally *MSR1* were up-regulated by LPS and/or GM-CSF [19].

#### Nr4a family members

Nr4a1 (Nur77), Nr4a2 (Nurr1), and Nr4a3 (NOR-1) are ligand-independent transcription factors in the nuclear hormone receptor superfamily whose expression is induced rapidly in a variety of cell types following a wide range of inflammatory or non-inflammatory stimuli [56], [57]. Expression of Nr4a family members is induced by inflammatory cytokines or oxidized lipids in murine macrophages [58], and by live bacteria or to a lesser extent LPS in murine mast cells [59]. Expression in neutrophils at the protein level has not been described, but all three NR4A family members were among the many transcription factors noted to have significant changes in gene expression in one study of human neutrophils, and these changes differed among the three stimuli used in vitro [22]. In another study, *NR4A3* was induced by either LPS or GM-CSF/IFNγ in vitro [19]. In our experiments, *Nr4a2* and *Nr4a3* were up-regulated only in SF neutrophils, and *Nr4a1* was up-regulated more in SF than TG neutrophils (**Fig. 2B**). These 3 genes were among the 49 genes with at least 2-fold higher expression in SF than in blood, TG, or UA neutrophils. Nr4a proteins have been shown to both induce and suppress expression of inflammatory genes [56]. Nr4a proteins play important roles in stimulating lipolysis and utilization of glucose [56], which is intriguing in light of the up-regulation of genes related to uptake and metabolism of lipids particularly in SF neutrophils.

#### Glutathione metabolism

Also notable was differential regulation of genes related to the synthesis, use, and recycling of glutathione, particularly in TG neutrophils (**Fig. 2C**). Increased capacity to synthesize glutathione is suggested by up-regulation of the genes for the rate-limiting enzyme, glutamate-cysteine ligase (*Gclc, Gclm*), extracellular enzymes that cleave plasma glutathione to provide a source of cyst(e)ine for cellular use (*Ggt1, Ggt5*), and the major transporter for cyst(e)ine (*Slc7a11*) [60]. In contrast, expression of several genes related to the oxidation-reduction cycle of glutathione and NADP (*G6pd2, G6pdx, Gpx1, Idh1*) was down-regulated in SF neutrophils. Glutathione is known to be important in multiple facets of neutrophil biology, e.g., production of cysteinyl-leukotrienes [61] and a range of activities dependent on microtubule assembly, such as chemotaxis, degranulation, and phagocytosis [62], but the details of regulation of glutathione synthesis and use have not been studied intensively in neutrophils. Interpretation of our data as a response to oxygen stress is supported by the finding that expression of genes for five other anti-oxidant enzymes (*Cat*, *Prdx1, Prdx6, Sod2*, and *Txnrd1*) was also increased specifically in TG neutrophils (**Fig. 2C**).

#### Arachidonic acid metabolites

Changes in genes related to arachidonic acid metabolites suggested increased synthesis of prostaglandins (upregulation of *Ptgs1/Cox1* and *Ptgs2/Cox2*) and decreased synthesis of leukotrienes (upregulation of *Dpep2* and *Ptgr1*, downregulation of *Mgst2*, *Ggt5*, and *Lta4h*). Particularly notable was upregulation of *Ptgs1/Cox1* and two leukotriene receptors (*Cysltr1* and *Ltb4r1*) specifically by UA (**Fig. 2D**), an expression pattern that was uncommon in the dataset overall. Neutrophils are known to produce both leukotrienes and prostaglandins in response to uric acid [63], [64], immune complexes [65], or microbes [66], [67]. Our data suggest that this pathway may be upregulated more by UA in the peritoneal cavity than by TG in the peritoneal cavity or by immune complexes in SF. In that setting, it is intriguing to note that in humans, gout (caused by UA crystals) appears to respond better to inhibitors of cyclooxygenases (the products of the *COX/PTGS* genes) than do other forms of inflammatory arthritis, but with the caveat that in mice, COX-1 inhibitors are effective in preventing arthritis using the same model used in this study [68].

#### Antigen processing and presentation

Genes for MHC class II molecules (*H2-Aa, H2-Ab1, H2-Eb1*) were significantly up-regulated only in SF neutrophils, and invariant chain (*Cd74*) was up-regulated in SF and TG neutrophils (**Fig. 2E**). Among co-stimulatory molecules, expression of *Cd80* appeared to be up-regulated in all 3 activated neutrophil populations. Genes for several proteases involved in production of peptide antigens (*Ctsb, Ctsl, Ctss*, *Lgmn*) and for a reducing agent important in antigen processing (*Ifi30/Gilt*) were also up-regulated particularly in SF neutrophils (**Fig. 2E**). Induction of genes related to antigen presentation has been noted to differ with different forms of activation of human neutrophils in vitro [19]. Mouse neutrophils co-incubated with T cells in vitro express MHC class II, CD80, and CD86 proteins and can process and present exogenous antigen to T cells [69]. In the same study, freshly isolated TG neutrophils did not express MHC class II proteins, consistent with our gene expression data. Thus, the conditions in SF may promote antigen presentation by neutrophils more effectively than peritonitis induced by TG or UA, but this hypothesis requires confirmation at the level of protein expression.

#### Apoptosis

Regulation of apoptosis in neutrophils has been a subject of intensive study. Apoptosis is the normal, non-inflammatory mechanism by which unstimulated neutrophils die after a short time in the circulation, and inhibition of apoptotic cell death is one of the salient features of neutrophil activation [14], [15], [70], [71]. Because of this literature, and because induction of anti-apoptotic genes has been commented upon in multiple previous studies of gene expression in activated neutrophils [17], [18], [23], [24], and because interpretation of gene expression patterns alone provides little insight into the activity of apoptotic pathways, we will only comment briefly on our data. Expression of several pro-apoptotic receptors (*Fas, Tnfrsf1b*), Bcl-2 family members (*Bcl2l11 = Bim*; *Bax*), and caspases (*Casp8*) was up-regulated, particularly in TG neutrophils (**Fig. 2F**). More striking was the up-regulation of anti-apoptotic Bcl-2 family members (*Bcl2l1 = Bcl-XL*; *Bcl2a1-4 = A1*) and other inhibitors (*Cflar, Xiap*), again predominantly in TG neutrophils (**Fig. 2F**). Consistent with the literature, among anti-apoptotic genes, *Bcl2* was not expressed, but *Mcl1*, known to be important in protection of neutrophils from apoptosis [70], [71], was expressed in all neutrophil populations at higher levels than in most other leukocytes (mean 6405+/−826, versus 2649+/−1182). Finally, the up-regulation of the glutathione pathway and anti-oxidant enzymes (see above) particularly in TG neutrophils can be interpreted as an anti-apoptotic response [72].

#### NFκB and its proximal regulators

Genes encoding the non-canonical NFκB subunits (*Nfkb2* and *Relb*) were up-regulated, as were genes encoding inhibitors of NFκB (*Nfkbia*, *d*, and *e*) and a kinase that inactivates these inhibitors in the non-canonical pathway (*Ikbke*, along with the regulatory subunit *Ikbkg/Nemo*), particularly in cells elicited with TG (**Fig. 2G**). These results suggest up-regulation of the non-canonical NFκB pathway.

Members of the canonical NFκB pathway are present in resting human neutrophils, and activation of this pathway in neutrophils activated by various stimuli has been described [30]. In the same study, the non-canonical isoforms NFκB2/p52 and RelB were not detected in resting cells, but it was not reported whether these isoforms were searched for after activation [30]. Thus, it is not clear that NFκB2/RelB involvement in activated neutrophils has been ruled out even in the specific setting of human cells stimulated in vitro, and increased expression of mRNA for one or both of these isoforms in stimulated human neutrophils has been found in multiple other studies [19]–[22]. Since much of the regulation of NFκB activity is post-transcriptional, it is difficult to draw conclusions on the basis of transcription patterns, other than to say that use of the non-canonical pathway is plausible. For example, McDonald et al. reported increased transcription of IκB-α (*Nfkbia*) after neutrophil activation, as has been seen in multiple other studies including this one [19], [20], [22], but noted that this increase occurred in response to the degradation of IκB-α protein [30]. Therefore, the change seen in mRNA for *Nfkbia* is biologically relevant, but interpretation is not straightforward in a system subject to feedback regulation.

#### Pathways for responding to microbial products

Signaling pathways from pattern recognition receptors (PRRs) for microbial products, including multiple Toll-like receptors (TLRs), NOD-like receptors (NLRs), and inflammasomes, are known to operate in neutrophils [73]–[75]. These pathways are also presumed or known to be relevant to the three inflammatory conditions being studied: autoantibody-induced arthritis is exacerbated by the TLR4 agonist lipopolysaccharide (LPS) [76], uric acid crystals deliver inflammatory signals via the NLRP-containing inflammasome [31], and although the pro-inflammatory components of thioglycollate broth have not been identified, the fact that it is a microbial extract makes it highly likely that multiple microbial products are involved.

Components of the NLR and inflammasome pathways were most prominently altered in TG neutrophils (**Fig. 2H**). Since both stimulatory (*Nod1, Birc3, Pycard/Asc, Hsp90*) and inhibitory (*Tnfaip3/A20, Mefv/Pyrin*) components were up-regulated, as were both pro-inflammatory (NFκB pathway, as above) and pro-apoptotic (*Casp8*) downstream effectors, the net biologic effects of these changes are difficult to predict. In contrast, multiple members of a pathway for sensing cytoplasmic DNA (*Zbp1/Dai, Tmem173/Mita, Ripk3/Rip3*) were equally up-regulated in TG and UA but not SF neutrophils. Changes in components of TLR pathways were more complex, but up-regulation of *Tlr4* and *Cd14* in multiple conditions, up-regulation of two components of the MyD88-independent pathway downstream of Tlr4 (*Traf3, Tbk1*) specifically in TG neutrophils, and down-regulation of the MyD88-dependent pathway component *Irak4* as well as a secreted LPS-binding protein (*Lbp*) specifically in SF neutrophils suggested that response to LPS is a particular object of differential regulation under different stimulating conditions (**Fig. 2H**).

Signaling from Tlr4 and other TLRs proceeds not only to NFκB and apoptotic pathways, but also to multiple interferon-inducible regulatory factors (IRFs), which we will discuss in more detail later since their importance was implicated by a separate analysis.

### Functions of genes down-regulated in activated neutrophils

Although down-regulated genes were nearly as numerous as up-regulated genes (see **Fig. S1**), they were not distributed as clearly into functional groups. Only 3 GO terms, redundant and consisting of multiple genes for histones, showed significant enrichment in any activating condition. Further analysis of histone genes revealed down-regulation of most genes in the replication-dependent histone clusters [77], most strikingly the genes for H3 isoforms, in all 3 activated populations. However, expression of replication-independent histone genes, particularly the “replacement variant” H3.3 genes *H3f3a* and *H3f3b*, was unchanged (**Fig. 2I**). Most likely, this finding simply reflects the fact that neutrophils do not divide, but it is also possible that neutrophils produce a unique complement of histones related to the production of neutrophil extracellular traps (NETs) [78], the anti-microbial properties of histones [79], or the toxic or regulatory interactions of extracellular histones with other cells [80], [81].

### Identification of regulatory genes likely to be important in neutrophil activation

One of the major products of ImmGen is the definition of modules of genes whose expression is correlated across leukocyte populations, with subsequent assignment of probable regulatory genes to each module [10]. In order to use this regulatory model to predict which regulators are important in neutrophil activation under different conditions, we first separated genes into 25 clusters of 1–128 genes, defined by similar patterns of expression, using K-means clustering of expression data from the individual replicates of SF, TG, UA, and blood neutrophils (**File S2**). The validity of this approach was supported by the fact that correlation coefficients (comparing individual genes to the mean expression profile for each cluster) were 0.86–0.94, whereas coefficients generated using randomized expression data did not exceed 0.75 (data not shown).

To identify regulators of interest, genes within pools of clusters (11 clusters of genes up-regulated versus blood, 9 clusters of down-regulated genes, and 5 clusters of genes both up- and down-regulated in different populations, to improve statistical power) were analyzed for distribution into ImmGen modules and for over-represented association with particular regulatory genes via those modules (see Methods). Importantly, these modules and assignment of regulatory genes were defined before any data from activated neutrophil populations were included in the ImmGen database (thus avoiding any bias), and the module definitions did not change after incorporation of these data.

**Figure 3:**
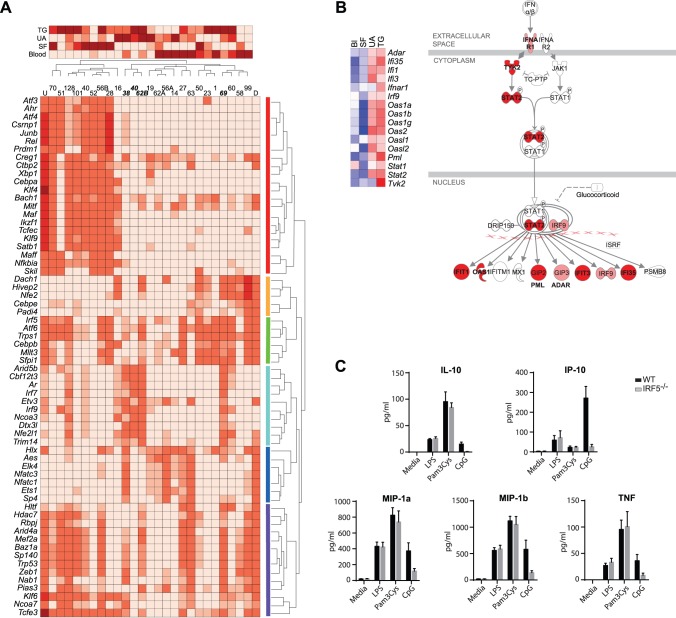
Regulatory genes implicated in neutrophil activation, with further focus on IRF family members. **A.** Genes were placed into 25 clusters (1–128 genes each, shown as the column headings; A and B are used to identify clusters that have the same numbers of genes) based on patterns of expression in individual samples of neutrophils from blood, SF, UA, and TG, as shown in the heatmap at the top. Clusters that clearly represented up-regulated (U) or down-regulated (D) genes (relative to blood) were pooled and were used to generate a list of predicted regulatory genes (rows) showing enrichment based on the ImmGen regulatory model. Association of each of the 64 regulators with each of the 25 gene clusters was then quantified (P-value of chi-square test), and this matrix of P-values was subjected to hierarchical clustering in order to identify related regulators (rows) and related gene clusters (columns). The lower heatmap indicates these P-values (darker = lower), and the dendrogram and colored bars on the right show groups of regulators with similar patterns of association with various gene clusters. The presence of patterns in the top heatmap (e.g., clustering of clusters characterized by up-regulation in TG, SF, or UA, or by down-regulation in SF), which shows normalized average expression in the 4 neutrophil populations in each cluster, validates this method. The group of regulators shown in light blue was associated with gene clusters indicated in bold; inspection of genes in these clusters led to implication of the type 1 interferon pathway and Irf9. **B.** Up-regulation of genes induced by type 1 interferons via Irf9, in TG and/or UA but not SF neutrophils. The heatmap shows mean expression in blood (BL), SF, UA, and TG neutrophils. Mean expression across all four conditions was placed at the center of the gradient (white) for each gene. The full color gradient for each gene represents an 8-fold difference in expression. The list of genes of interest and the pathway diagram were generated using the KEGG and Ingenuity databases. Only genes showing at least 1.5-fold differences in expression comparing conditions are shown in the heatmap. In the pathway diagram, genes showing statistically significant (Q<0.05 by ANOVA) differences that varied 2-fold in at least one pairwise comparison of conditions are shown in red, and genes showing fold differences of 1.5–2 and/or not meeting statistical significance are shown in pink. **C.** *Irf5* is required for production of several cytokines and chemokines by mouse neutrophils stimulated in vitro with the TLR9 ligand CpG-B, but not for production induced by the TLR2 ligand Pam3Cys nor the TLR4 ligand LPS. The panels show the mean ± SEM of 3 independent experiments using FACS-sorted Gr1^hi^CD11b^+^F4/80^−^ neutrophils. Since secretion varied between experiments but reliably did so in parallel for the different analytes, data were analyzed by determining the fold difference between *Irf5−/−* and WT in each experiment and applying one-sample T-tests to the fold-differences for the 3 experiments. P values for cells treated with CpG were 0.014 for TNF and <0.01 for the other proteins, and 0.13–0.97 for other TLR ligands.

Sixty-four regulators were implicated using this approach. The degree of over-representation of regulated genes in each of the 25 individual gene clusters was then determined (P-value of chi-square test), and these data were used to create a matrix of P-values for each regulator with each cluster. Hierarchical clustering of this matrix was informative (**Fig. 3A**). Clusters of up- and down-regulated genes clustered independently of each other, with the 5 clusters of more complex patterns mixed in. Among clusters of up-regulated genes, there was some clustering of patterns characterized by particularly high expression in TG neutrophils, or SF neutrophils, or both TG and UA but not SF neutrophils. These results confirmed that implicated regulators were shared across related expression patterns. Twenty-two regulators were prominently associated with multiple clusters of up-regulated genes and few if any other patterns; conversely, 5 regulators were strongly associated primarily with down-regulated genes. Twenty regulators were associated with many clusters with a variety of patterns and thus were implicated in both up- and down-regulation of genes. All of these 47 regulators appeared to be associated with changes in gene expression across all 3 activating conditions.

In contrast, the remaining 17 regulators were associated with combinations of clusters that shared patterns specific to activating conditions. Seven regulators were associated mostly with down-regulated genes, but particularly for genes down-regulated in SF. Ten regulators were associated with 5 clusters in which gene expression was up-regulated in TG and UA, but not SF. Four of these 5 clusters showed convincing association with the 10 regulators and were examined to see whether additional functional pathways could be identified.

### Interferon regulatory factors (IRFs) in activated neutrophils

The list of 203 genes that were up-regulated in TG and UA but not SF and that were implicated in a shared regulatory network was analyzed using DAVID. After adjustment for multiple comparisons, no GO category showed significant enrichment. The chemokine signaling pathway in KEGG was significantly enriched (Q = 0.03), but the 9 genes in this pathway included several that are involved in many pathways (e.g., *Akt3, Nfkb1, Stat2*), so this result was not particularly informative. In contrast, inspection of the list showed multiple genes for oligoadenylate synthases (*Oas1a, Oas1g, Oas2*, and *Oasl2*), and genes regulating expression of *Oas* or co-regulated with *Oas* were found to have similar patterns of expression (**Fig. 3B**). This result supports the hypothesis that Irf9, probably induced via the type I interferon receptor, plays a role in up-regulation of genes in TG and UA but not SF neutrophils. Irf9 has not been implicated in neutrophil function previously.

Two other IRFs were among the 64 regulators implicated in neutrophil activation. *Irf7* was in the same group of 10 regulators as was *Irf9*, associated with up-regulation of genes by TG and UA but not SF (see **Fig. 3A**). Several genes that we had identified as being up-regulated in the TLR signaling and cytosolic DNA-sensing pathways (see **Fig. 2H**) encode members of pathways that activate Irf7 or induce *Irf7*, adding to the plausibility that Irf7 plays a role in neutrophils activated via the pathways of innate immunity. *Irf7* was expressed at similar levels in both unstimulated and activated neutrophils, but Irf7 activity is also regulated by post-translational modifications, including phosphorylation (see **Fig. 2H**) [82].

*Irf5*, in contrast, was implicated in both up- and down-regulation of genes in all 3 activating conditions (see **Fig. 3A**). Expression of *Irf5* mRNA was easily detectable in blood neutrophils (mean 495) and increased significantly in SF (mean 909, P<0.0001), UA (mean 1204, P = 0.03), and TG (mean 1727, P<0.0001) neutrophils. Irf5 has diverse functions that include the induction of type I interferons and proinflammatory cytokines following viral infection or downstream of Toll-like receptors (TLRs) and nucleotide-binding oligomerization domain 2 (NOD2) [34], [83]–[87], and participation in apoptotic pathways induced by viral infection, DNA damage, Fas-ligand, or tumor necrosis factor-related apoptosis inducing ligand (TRAIL) [86], [88], [89]. However, Irf5 has not previously been implicated in neutrophil biology. To determine whether Irf5 plays a role in neutrophil function, we isolated bone marrow neutrophils from IRF5-deficient (*Irf5*−/−) and wild-type (WT) mice and compared their secretion of cytokines and chemokines in vitro in response to ligands for different TLRs. Fifteen candidate cytokines/chemokines were originally chosen on the basis of gene expression >75 in a neutrophil population in ImmGen, inclusion in an Irf5-regulated ImmGen module, inclusion in a cluster predicted to be regulated by Irf5 in the current experiments, and/or known production by human neutrophils in vitro [13]; secretion of IL-1α, IL-12(p40), IL-15, MCP-1, M-CSF, and MIG was not detectable in our model system in a preliminary experiment, so only 9 mediators were studied further.

*Irf5*−/− neutrophils (CD11b^+^Gr1^hi^F4/80^−^) secreted less IL-10, IP-10, MIP-1α, MIP-1β, and TNF-α than WT neutrophils in response to a TLR9 agonist (**Fig. 3C**). Analogous results with lower absolute amounts were obtained in a single experiment using FACS-sorted Ly6G^hi^CD11b^+^ neutrophils (data not shown). In contrast, *Irf5*−/− and WT neutrophils secreted comparable amounts of these proteins in response to TLR2 or TLR4 agonists, demonstrating that the difference in TLR9-induced responses between WT and *Irf5*−/− neutrophils was not due to a generalized inability of the *Irf5*−/− neutrophils to respond (**Fig. 3C**). Conversely, G-CSF was detectable after treatment with the TLR9 agonist in this model system and did not differ between *Irf5−/−* and WT neutrophils (data not shown), indicating that the effect of the TLR9 agonist on other cytokines did not simply indicate non-specific toxicity. No differences were seen in secretion of IL-1β, KC, or MIP-2 between *Irf5−/−* and WT neutrophils with any TLR agonist (data not shown, P values 0.076–0.66).

Although our primary aim was simply to see whether secretion of cytokines/chemokines by neutrophils stimulated through any TLR was Irf5-dependent, and thereby to provide functional validation of the importance of Irf5 as a novel regulator of neutrophil function implicated via the ImmGen regulatory model, discussion of the relevant literature is warranted. Dependence of TLR-induced inflammatory cytokine production upon Irf5 has varied widely with the cell types studied [34], [87], [90], [91], but there is definitely precedent for TLR9-induced secretion of TNF being reduced in mouse macrophages or dendritic cells lacking Irf5 [34], [91]. Transfection of *IRF5* into a human B lymphoma cell line increased production of MIP-1α, MIP-1β, IP-10, and other chemokines after stimulation by viruses in vitro [83].

Seemingly in contrast to our data, forced expression of *IRF5* in human macrophages decreased production of IL-10, and bone-marrow-derived macrophages from *Irf5−/−* mice secreted more IL-10 than did cells from wild type mice [92], but the cell types and model systems differed from those used in our experiments. Finally, Zhang et al. called into question many earlier reports of cytokine secretion by neutrophils by using data from neutrophils purified using antibodies to Ly6G rather than Gr-1 (which binds both Ly6C and Ly6G) [39]. We used negative selection of cells staining for F4/80 and bright staining for Gr-1, rather than simply positive staining for Gr-1, and therefore undoubtedly achieved better purification than in some early papers. However, the results we obtained in a single experiment using Ly6G sorted cells are also quite similar to those obtained by Zhang et al.; we agree that the absolute amounts of TNF are small on a per-cell basis, but our goal was to determine whether such secretion was Irf5-dependent rather than to compare it to the much larger amount made by macrophages.

## Summary and Conclusions

Neutrophils exhibit a pattern of gene expression distinct from that of other mouse leukocytes, with that distinction determined at least as much by genes that neutrophils down-regulate (e.g., genes related to translation) as by genes that they up-regulate. Nevertheless, a moderate number of genes were relatively neutrophil-specific and continued to be expressed after neutrophil activation, and most of these genes, such as *Stfa2l1* and *Mrgpr2a* and *b*, are of unknown function. The major caveat to this interpretation is that gene expression patterns in eosinophils have not yet been reported in ImmGen or any other comprehensive database.

Numerous changes in gene expression were seen after neutrophil activation in vivo, particularly in peritoneal neutrophils elicited with TG compared to peritoneal neutrophils elicited with UA or SF neutrophils elicited with immune complexes. Most of the differences between these three stimuli were quantitative rather than qualitative. For example, changes in genes for lysosome components and genes related to apoptosis were seen with all stimuli but were greater in magnitude in TG neutrophils.

However, certain pathways were more specific to particular stimuli. Genes related to the non-canonical NFκB pathway and to the synthesis and use of glutathione were up-regulated in TG neutrophils. Genes related to antigen processing and presentation, uptake of modified lipoproteins, and the Nr4a family of transcription factors were up-regulated in SF neutrophils. Receptors for leukotrienes were up-regulated in UA neutrophils.

Finally, a regulatory model derived from ImmGen was used to infer the involvement of many transcription factors and other regulatory genes in up- and/or down-regulation of genes during neutrophil activation. For example, Irf7 and Irf9 were implicated in up-regulating a group of genes with increased expression in TG or UA but not SF neutrophils. Irf5 was implicated in both up- and down-regulation of many genes after all stimuli, and a novel role for Irf5 in optimal induction of secretion of cytokines and chemokines by a TLR9 agonist in neutrophils was confirmed using *Irf5−/−* mice.

Three technical points must be discussed in considering the validity of our data and their interpretation. First, since monocytes contain 10–20 times as much mRNA per cell as do neutrophils, 1–2% contamination could yield RNA that is 10–30% of monocyte origin, so the possibility of monocyte/macrophage contamination must be addressed in any study of gene expression in neutrophils [13]. The finding that many genes were not expressed in neutrophils but were expressed in all other leukocyte populations argues against such contamination. In addition, a plot of gene expression in macrophages versus TG-activated neutrophils showed a poor correlation, leading to the conclusion that only 5 genes that were expressed at extremely high levels in macrophages might give strong enough signals via contamination to produce modestly elevated levels in TG neutrophils (data not shown). Second, it is possible that some changes in gene expression among neutrophils isolated from local sites actually derive from circulating mediators rather than being elicited at the site of inflammation. Arguing against this interpretation, the gene expression pattern in bone-marrow neutrophils from arthritic mice was very similar to expression in bone-marrow neutrophils from normal mice (data not shown, and see **Fig. 1B**). Third, we are unable to address the possibility that some of the differences seen comparing SF to TG or UA neutrophils resulted from the time course (7 days versus 18 hours) rather than the stimuli, since there is no common time point feasible for collection of neutrophils in all of these models.

The strengths of this study include the use of rigorous, standardized protocols for collection of cells and data, both for neutrophils and other leukocytes; the resulting ability to compare neutrophils to numerous other leukocyte populations; and the comparison of neutrophils activated in different ways in vivo. We endeavored to begin analyses in an unbiased manner free of hypotheses and to report all results regardless of novelty, so as to establish a broad framework upon which we and others could use this data-set as a resource for future hypothesis-driven experiments.

The obvious limitation of this study is that most observations were not confirmed at the level of protein expression or proof of functional significance, the one exception being the demonstration of the importance of neutrophil expression of Irf5 in optimal secretion of multiple cytokines and chemokines. In addition, our study would have been stronger if we had been able to include a model of active bacterial infection. Since we must defend this study in part as a hypothesis-generating exercise, it may be most appropriate to end the discussion with some hypotheses:

- Some of the few genes that are highly specific to neutrophils, such as *Stfa2l1* and *Mrgpr2a* and *b*, will be found to be essential for functions unique to neutrophils, such as NETosis or other anti-microbial functions yet to be discovered.
- Proper regulation of anti-oxidant pathways and cellular energetics, in part regulated by Nr4a family members, will be found to be necessary for a neutrophil function essential to the orderly development and resolution of acute inflammation, namely, promoting neutrophil cell death at the right time and by the right mechanism(s).
- IRF family members will be important for induction of anti-microbial and inflammatory mediators in neutrophils via innate immune receptors.
- The limited capacity for translation in the mature neutrophil will reveal a weak correlation between the amount of mRNA and the amount of new protein produced, leading to new insights into regulation of translation.

## Supporting Information

Figure S1 Changes in gene expression in neutrophils activated in vivo by different stimuli: synovial fluid (SF), thioglycollate (TG), or uric acid (UA). **A.** Comparison of fold-changes in gene expression relative to circulating neutrophils, among all 1283 genes showing significant variation across all conditions by ANOVA. Non-transformed data are shown on a log scale. Log-transformed data on a linear scale were used to calculate correlation coefficients (r) and slopes. The slope in the middle panel (1.08; 95% confidence interval 1.04–1.13) indicates higher expression in TG than UA. **B.** Venn diagrams showing the numbers of genes up- or down-regulated in the three activating conditions. Top: fold-change >2 was used as the cut-off for all conditions. Bottom: conditions were relaxed so that if one condition had fold change >2, the others could have fold change >1.5.
(EPS)

Table S1 Validation of neutrophil-specific gene expression by RNA-Seq of major leukocyte populations.
(DOCX)

Table S2 Genes with increased expression relatively specific to a stimulating condition.
(DOCX)

Text S1 Comparison of neutrophils activated by different stimuli.
(DOCX)

File S1 Expression, in purified leukocyte populations in the ImmGen database, of genes comprising a neutrophil signature in the BioGPS database [47]. All genes in the BioGPS signature for which there were comparable data in ImmGen are shown. Blue indicates relatively low expression, red indicates high expression; expression data were log-transformed and mean-centered for each gene (i.e., row-normalized) using the HeatMapImage module of GenePattern. Gene names are shown on the right, hierarchical clustering of the expression patterns on the left, and ImmGen populations (not clustered) along the top; text can be viewed using a photo viewer with a magnification function. Neutrophil-related populations at the left edge include common myeloid precursor (SC_CMP_BM), granulocyte-monocyte precursor (SC_GMP_BM), bone-marrow neutrophils (GN_BM), blood neutrophils (GN_Bl), neutrophils from inflamed synovial fluid (GN_Arth_SynF), uric-acid-induced peritoneal neutrophils (GN_UrAc_PC), and thioglycollate-induced peritoneal neutrophils (GN_Thio_PC).
(PNG)

File S2 Gene expression in mouse neutrophils. Expression of all 1283 probes that passed filters for analysis (see Methods) is shown, both in individual samples and as means of 3–4 replicate samples. Assignment to clusters based on similar patterns of expression across 4 conditions is shown in the final 2 columns: i) using our original notation, in order to show where clusters produced by ExpressCluster were pooled after inspection, and ii) using notation used in Figure 3A, which provides the numbers of probes in each cluster.
(XLSX)
