# Supplementary material for: Gene Expression during the Generation and Activation of Mouse Neutrophils: Implication of Novel Functional and Regulatory Pathways
Source: PLoS One. 2014 Oct 3;9(10):e108553. doi: 10.1371/journal.pone.0108553 (PMC4184787; doi:10.1371/journal.pone.0108553)
Supplement: Table S1 — Validation of neutrophil-specific gene expression by RNA-Seq of major leukocyte populations. (DOCX) [file pone.0108553.s002.docx]

**Table S1**. Validation of neutrophil-specific gene expression by RNA-Seq of major leukocyte populations.

| \| Gene Symbol \| GN \| DC \| B1ab.PC \| NK \| Treg \| CD4 T \| CD8 T \| B \| Tgd \| \| --- \| --- \| --- \| --- \| --- \| --- \| --- \| --- \| --- \| --- \| \| 1100001G20Rik \| 7312.295 \| 6.308789 \| 1.413274 \| 0.835286 \| 2.345701 \| 0.899818 \| 3.257361 \| 177.8354 \| 9.692802 \| \| 2010002M12Rik \| 2211.833 \| 25.23515 \| 21.90574 \| 21.71743 \| 44.56833 \| 62.98725 \| 57.54672 \| 39.60201 \| 11.63136 \| \| 9830107B12Rik \| 4068.031 \| 5.257324 \| 10.59955 \| 5.011714 \| 23.45701 \| 6.298725 \| 12.48655 \| 9.7137 \| 5.815681 \| \| Ankrd22 \| 892.717 \| 0 \| 0 \| 0.835286 \| 0 \| 1.799636 \| 0 \| 0 \| 0 \| \| Arg2 \| 16411.84 \| 19.97783 \| 5.653094 \| 6.682286 \| 11.72851 \| 5.398907 \| 3.800255 \| 4.483246 \| 0 \| \| Ceacam10 \| 1672.937 \| 3.154394 \| 0 \| 0 \| 1.172851 \| 0 \| 0.542894 \| 0.747208 \| 1.93856 \| \| Chi3l1 \| 18340.62 \| 12.61758 \| 5.653094 \| 3.341143 \| 3.518552 \| 3.599271 \| 7.057616 \| 11.20811 \| 19.3856 \| \| Clec5a \| 13205.68 \| 64.13935 \| 2.11991 \| 5.011714 \| 2.345701 \| 3.599271 \| 2.171574 \| 5.230454 \| 1.93856 \| \| Csf3r \| 635821.3 \| 1059.876 \| 200.6848 \| 186.2687 \| 255.6815 \| 217.7559 \| 218.7861 \| 203.2405 \| 178.3475 \| \| Cxcr2 \| 119664 \| 252.3515 \| 149.1004 \| 70.99929 \| 145.4335 \| 122.3752 \| 98.80663 \| 85.92888 \| 135.6992 \| \| Dhrs9 \| 12086.15 \| 90.42597 \| 2.11991 \| 7.517571 \| 4.691403 \| 4.499089 \| 4.886042 \| 2.241623 \| 3.877121 \| \| Fam123a \| 2425.94 \| 0 \| 0 \| 0 \| 0 \| 3.599271 \| 1.628681 \| 0.747208 \| 0 \| \| Grina \| 70502.87 \| 1277.53 \| 1198.456 \| 3029.581 \| 1013.343 \| 1303.836 \| 1000.553 \| 1030.399 \| 883.9835 \| \| Il1f9 \| 26672.64 \| 11.56611 \| 13.4261 \| 9.188143 \| 10.55566 \| 16.19672 \| 14.11523 \| 9.7137 \| 1.93856 \| \| Kctd11 \| 2708.997 \| 338.5717 \| 154.7534 \| 1059.978 \| 100.8652 \| 48.59016 \| 66.23302 \| 171.8578 \| 348.9409 \| \| Mgam \| 10055.77 \| 5.257324 \| 0.706637 \| 4.176429 \| 41.04978 \| 0 \| 4.886042 \| 3.736038 \| 0 \| \| Mir29c \| 0 \| 0 \| 0 \| 0 \| 0 \| 0 \| 0 \| 0 \| 0 \| \| Mrgpra2a \| 83.46541 \| 0 \| 0 \| 0 \| 0 \| 0 \| 0 \| 0 \| 0 \| \| Mrgpra2b \| 390.1101 \| 0 \| 0 \| 0 \| 1.172851 \| 0 \| 0 \| 0 \| 0 \| \| Ppp1r3d \| 9386.229 \| 58.88203 \| 4.239821 \| 5.847 \| 7.037104 \| 4.499089 \| 3.800255 \| 2.988831 \| 3.877121 \| \| Prrg2 \| 1328.189 \| 202.9327 \| 43.10484 \| 49.28186 \| 14.07421 \| 17.99636 \| 17.91549 \| 48.5685 \| 17.44704 \| \| Rlf \| 14991.11 \| 1579.3 \| 1808.283 \| 1475.115 \| 1721.745 \| 1555.785 \| 1745.403 \| 1380.84 \| 1806.738 \| \| Rnf11 \| 16152.37 \| 1468.896 \| 1352.503 \| 2028.074 \| 1101.307 \| 992.4991 \| 877.8589 \| 1152.194 \| 1417.088 \| \| S100a7a \| 30.84591 \| 6.308789 \| 0 \| 0.835286 \| 0 \| 0 \| 0 \| 0 \| 0 \| \| Sfxn5 \| 18543.84 \| 128.2787 \| 130.7278 \| 67.65814 \| 59.81539 \| 42.29144 \| 47.23174 \| 143.4639 \| 31.01697 \| \| Sgms2 \| 15617.1 \| 85.16865 \| 66.42385 \| 34.24671 \| 123.1493 \| 68.38615 \| 70.57616 \| 29.88831 \| 143.4535 \| \| Slc22a20 \| 101.6101 \| 0 \| 0 \| 0 \| 0 \| 0 \| 0 \| 0 \| 0 \| \| Slfn4 \| 194868.1 \| 187.1607 \| 98.22251 \| 45.94071 \| 127.8407 \| 92.68124 \| 85.77718 \| 64.25986 \| 73.66529 \| \| Spatc1 \| 342.934 \| 0 \| 0 \| 0 \| 0 \| 0 \| 0 \| 0 \| 0 \| \| Stfa2l1 \| 3362.204 \| 3.154394 \| 0.706637 \| 0.835286 \| 0 \| 0.899818 \| 0.542894 \| 3.736038 \| 1.93856 \| |  |  |  |  |  |  |  |  |  |
| --- | --- | --- | --- | --- | --- | --- | --- | --- | --- | --- | --- | --- | --- | --- | --- | --- | --- | --- | --- | --- | --- | --- | --- | --- | --- | --- | --- | --- | --- | --- | --- | --- | --- | --- | --- | --- | --- | --- | --- | --- | --- | --- | --- | --- | --- | --- | --- | --- | --- | --- | --- | --- | --- | --- | --- | --- | --- | --- | --- | --- | --- | --- | --- | --- | --- | --- | --- | --- | --- | --- | --- | --- | --- | --- | --- | --- | --- | --- | --- | --- | --- | --- | --- | --- | --- | --- | --- | --- | --- | --- | --- | --- | --- | --- | --- | --- | --- | --- | --- | --- | --- | --- | --- | --- | --- | --- | --- | --- | --- | --- | --- | --- | --- | --- | --- | --- | --- | --- | --- | --- | --- | --- | --- | --- | --- | --- | --- | --- | --- | --- | --- | --- | --- | --- | --- | --- | --- | --- | --- | --- | --- | --- | --- | --- | --- | --- | --- | --- | --- | --- | --- | --- | --- | --- | --- | --- | --- | --- | --- | --- | --- | --- | --- | --- | --- | --- | --- | --- | --- | --- | --- | --- | --- | --- | --- | --- | --- | --- | --- | --- | --- | --- | --- | --- | --- | --- | --- | --- | --- | --- | --- | --- | --- | --- | --- | --- | --- | --- | --- | --- | --- | --- | --- | --- | --- | --- | --- | --- | --- | --- | --- | --- | --- | --- | --- | --- | --- | --- | --- | --- | --- | --- | --- | --- | --- | --- | --- | --- | --- | --- | --- | --- | --- | --- | --- | --- | --- | --- | --- | --- | --- | --- | --- | --- | --- | --- | --- | --- | --- | --- | --- | --- | --- | --- | --- | --- | --- | --- | --- | --- | --- | --- | --- | --- | --- | --- | --- | --- | --- | --- | --- | --- | --- | --- | --- | --- | --- | --- | --- | --- | --- | --- | --- | --- | --- | --- | --- | --- | --- | --- | --- | --- | --- | --- | --- | --- | --- | --- | --- | --- | --- | --- | --- | --- | --- | --- | --- | --- | --- | --- | --- | --- | --- | --- | --- | --- | --- | --- | --- |
| Numbers indicate gene expression using RNA-Seq data (see Methods) from leukocyte populations from one mouse: splenic neutrophils (GN), dendritic cells (DC), NK cells, Treg cells, CD4+ T cells, CD8+ T cells, B cells, and γδ T cells (Tgd), and peritoneal B1 cells (B1ab.PC). |  |  |  |  |  |  |  |  |  |
|  |  |  |  |  |  |  |  |  |  |
|  |  |  |  |  |  |  |  |  |  |
|  |  |  |  |  |  |  |  |  |  |
|  |  |  |  |  |  |  |  |  |  |
|  |  |  |  |  |  |  |  |  |  |
|  |  |  |  |  |  |  |  |  |  |
|  |  |  |  |  |  |  |  |  |  |
|  |  |  |  |  |  |  |  |  |  |
|  |  |  |  |  |  |  |  |  |  |
|  |  |  |  |  |  |  |  |  |  |
|  |  |  |  |  |  |  |  |  |  |
|  |  |  |  |  |  |  |  |  |  |
|  |  |  |  |  |  |  |  |  |  |
|  |  |  |  |  |  |  |  |  |  |
|  |  |  |  |  |  |  |  |  |  |
|  |  |  |  |  |  |  |  |  |  |
|  |  |  |  |  |  |  |  |  |  |
|  |  |  |  |  |  |  |  |  |  |
|  |  |  |  |  |  |  |  |  |  |
|  |  |  |  |  |  |  |  |  |  |
|  |  |  |  |  |  |  |  |  |  |
|  |  |  |  |  |  |  |  |  |  |
|  |  |  |  |  |  |  |  |  |  |
|  |  |  |  |  |  |  |  |  |  |
|  |  |  |  |  |  |  |  |  |  |
|  |  |  |  |  |  |  |  |  |  |
|  |  |  |  |  |  |  |  |  |  |
