# Supplementary material for: Gene Expression during the Generation and Activation of Mouse Neutrophils: Implication of Novel Functional and Regulatory Pathways
Source: PLoS One. 2014 Oct 3;9(10):e108553. doi: 10.1371/journal.pone.0108553 (PMC4184787; doi:10.1371/journal.pone.0108553)
Supplement: Table S2 — Genes with increased expression relatively specific to a stimulating condition. (DOCX) [file pone.0108553.s003.docx]

**Table S2**. Genes with increased expression relatively specific to a stimulating condition.

| Specificity | Gene Symbol | BL | SF | UA | TG | SF/UA | SF/TG |
| --- | --- | --- | --- | --- | --- | --- | --- |
| SF | Nr4a3 | 56.66206 | 721.7091 | 66.02848 | 94.93068 | 10.93027 | 7.602485 |
|  | Apoe | 786.289 | 2036.868 | 342.1836 | 370.6233 | 5.95256 | 5.495791 |
|  | Pltp | 119.73 | 730.024 | 133.367 | 122.5832 | 5.4738 | 5.955336 |
|  | Rgs1 | 40.67892 | 165.6267 | 26.02699 | 35.15088 | 6.363652 | 4.711879 |
|  | Pdpn | 132.0023 | 648.5797 | 152.9329 | 155.5175 | 4.240942 | 4.170462 |
|  | Per1 | 472.781 | 1126.647 | 280.9803 | 297.5619 | 4.009699 | 3.78626 |
|  | Isy1 | 123.8802 | 673.1655 | 180.0403 | 168.9889 | 3.738971 | 3.98349 |
|  | H2-Aa | 49.4517 | 526.853 | 41.95484 | 149.7435 | 12.55762 | 3.518369 |
|  | Cdkn1a | 61.76441 | 403.7781 | 112.0347 | 116.333 | 3.604045 | 3.470882 |
|  | Egr1 | 244.6308 | 1030.714 | 131.5272 | 300.6483 | 7.836513 | 3.428306 |
|  | Ms4a6d | 35.58476 | 289.384 | 49.14724 | 85.41668 | 5.888102 | 3.387909 |
|  | Slc38a1 | 54.78563 | 443.6234 | 76.86411 | 137.6517 | 5.771528 | 3.222797 |
|  | Rhov | 76.61679 | 247.5278 | 46.67872 | 79.55799 | 5.302798 | 3.111288 |
|  | Dab2 | 47.56615 | 272.2262 | 59.26486 | 88.40509 | 4.593383 | 3.079304 |
|  | Sepn1 | 910.6788 | 2009.243 | 653.0447 | 608.3365 | 3.076732 | 3.302848 |
|  | C1qb | 67.90285 | 327.253 | 74.07878 | 106.5903 | 4.417634 | 3.070196 |
|  | Csrnp1 | 601.7486 | 1794.839 | 589.9633 | 538.8977 | 3.04229 | 3.330575 |
|  | Scd2 | 66.35001 | 203.7515 | 54.49784 | 71.07223 | 3.738709 | 2.866824 |
|  | Cnnm2 | 131.7477 | 350.3151 | 122.7743 | 96.98934 | 2.853327 | 3.611893 |
|  | H2-Ab1 | 67.62627 | 401.0013 | 64.71304 | 144.9746 | 6.196607 | 2.76601 |
|  | Ctsl | 234.4781 | 1216.591 | 269.1862 | 441.6534 | 4.519513 | 2.754627 |
|  | Nr4a2 | 79.00172 | 214.1529 | 80.20812 | 63.49715 | 2.669965 | 3.372638 |
|  | Emd | 290.5517 | 703.69 | 196.2138 | 268.6973 | 3.586342 | 2.618895 |
|  | Scpep1 | 52.41081 | 235.3738 | 50.73755 | 90.08043 | 4.639044 | 2.612929 |
|  | C1qc | 57.45023 | 188.1711 | 58.24357 | 72.6416 | 3.230762 | 2.590404 |
|  | Slc38a1 | 38.8186 | 153.2515 | 45.51812 | 59.88138 | 3.366825 | 2.559252 |
|  | Slc7a8 | 53.71884 | 226.8652 | 59.73759 | 90.25263 | 3.797695 | 2.513668 |
|  | Itgb5 | 79.4058 | 201.3272 | 66.7255 | 82.33601 | 3.017245 | 2.44519 |
|  | 2310016C08Rik | 52.09378 | 450.9801 | 70.81268 | 184.441 | 6.368635 | 2.445118 |
|  | Nt5e | 260.9826 | 903.327 | 206.0646 | 374.4625 | 4.383708 | 2.41233 |
|  | Naglu | 114.7551 | 336.1196 | 127.4174 | 142.4228 | 2.637942 | 2.360013 |
|  | H2-Eb1 | 121.9531 | 455.053 | 132.5572 | 193.3362 | 3.432881 | 2.353688 |
|  | Cxcl16 | 51.88811 | 138.4675 | 49.37317 | 59.01513 | 2.804509 | 2.346305 |
|  | Isca1 | 277.3442 | 602.1553 | 257.56 | 245.7537 | 2.337922 | 2.450239 |
|  | Rabgef1 | 204.3953 | 830.3936 | 221.8837 | 355.7661 | 3.742473 | 2.3341 |
|  | Snapc1 | 72.84287 | 175.0875 | 74.30143 | 79.6098 | 2.356449 | 2.199321 |
|  | Zfand2a | 233.3309 | 950.6064 | 331.8003 | 435.2846 | 2.864996 | 2.183874 |
|  | Cdk4 | 108.6715 | 271.8373 | 108.4964 | 124.8911 | 2.505496 | 2.176594 |
|  | Lrp1 | 121.8023 | 463.6265 | 214.1542 | 200.0795 | 2.164919 | 2.317211 |
|  | Anpep | 52.41974 | 129.4464 | 52.40484 | 59.89959 | 2.470123 | 2.161057 |
|  | Isca1 | 284.3437 | 570.4477 | 263.1299 | 265.4606 | 2.167932 | 2.148897 |
|  | Btbd19 | 229.1089 | 566.0925 | 160.3133 | 265.29 | 3.531163 | 2.133863 |
|  | Gla | 249.2097 | 2044.43 | 322.6283 | 965.8814 | 6.336795 | 2.116647 |
|  | Acot1 | 108.0997 | 345.8213 | 166.0756 | 157.44 | 2.082313 | 2.196527 |
|  | Nr4a1 | 591.5923 | 3118.85 | 616.276 | 1504.609 | 5.060801 | 2.072865 |
|  | Errfi1 | 235.2085 | 550.7363 | 268.1939 | 266.6482 | 2.053501 | 2.065404 |
|  | Nceh1 | 158.6949 | 487.1287 | 103.9178 | 239.9683 | 4.687634 | 2.02997 |
|  | S100a10 | 75.76982 | 600.8738 | 230.3701 | 296.5127 | 2.608297 | 2.026469 |
|  | Tinf2 | 245.3115 | 520.5277 | 259.6712 | 192.8597 | 2.004565 | 2.698996 |
|  |  |  |  |  |  | TG/SF | TG/UA |
| TG | Nqo1 | 48.10247 | 53.4481 | 42.72504 | 777.3964 | 14.54488 | 18.19533 |
|  | 9030625A04Rik | 50.30266 | 78.41125 | 97.12637 | 718.9236 | 9.168627 | 7.40194 |
|  | Abcc1 | 84.24088 | 281.8957 | 110.4918 | 1896.486 | 6.727615 | 17.16404 |
|  | Mir146 | 10.14279 | 21.72492 | 39.19077 | 238.3148 | 10.96965 | 6.08089 |
|  | Ikbke | 81.28427 | 156.2245 | 593.0137 | 3058.657 | 19.5786 | 5.157818 |
|  | Gclm | 275.0742 | 319.2395 | 294.5552 | 1530.979 | 4.795705 | 5.197595 |
|  | Il2rg | 251.0553 | 710.9407 | 624.8546 | 3382.998 | 4.758482 | 5.414057 |
|  | Gdap10 | 226.8225 | 235.4575 | 345.0846 | 1583.934 | 6.727049 | 4.589989 |
|  | Ak2 | 322.4111 | 174.0955 | 193.8438 | 888.8407 | 5.105477 | 4.585346 |
|  | Zc3h12c | 32.28205 | 41.20292 | 37.02835 | 187.3556 | 4.547144 | 5.059789 |
|  | Icam1 | 169.9996 | 339.1693 | 376.3706 | 1642.752 | 4.843457 | 4.36472 |
|  | Eid3 | 73.08541 | 107.597 | 85.53653 | 460.1167 | 4.276299 | 5.379184 |
|  | Sod2 | 114.8482 | 254.319 | 192.6838 | 1003.876 | 3.947311 | 5.209968 |
|  | Gbe1 | 376.5854 | 281.118 | 226.3718 | 1040.3 | 3.700581 | 4.595537 |
|  | Cyb5 | 120.3454 | 94.18146 | 92.40373 | 343.5869 | 3.648137 | 3.718322 |
|  | Ggh | 133.6162 | 124.3666 | 213.5317 | 716.7978 | 5.763589 | 3.356868 |
|  | Prdx1 | 33.22358 | 117.4934 | 34.28349 | 392.9457 | 3.344407 | 11.46166 |
|  | Gm13139 | 38.72579 | 60.70281 | 50.99631 | 201.8856 | 3.325804 | 3.958828 |
|  | Zc3h12c | 43.4895 | 60.80917 | 64.40804 | 214.1129 | 3.521062 | 3.324319 |
|  | Agpat4 | 71.81557 | 212.8045 | 206.8537 | 699.7654 | 3.288301 | 3.3829 |
|  | Gss | 139.4467 | 146.7401 | 117.0372 | 453.5532 | 3.09086 | 3.87529 |
|  | Hivep2 | 24.29062 | 72.24777 | 74.84643 | 229.4453 | 3.175811 | 3.065548 |
|  | Nampt | 734.3102 | 1116.775 | 861.1635 | 3316.908 | 2.970077 | 3.851658 |
|  | Tbc1d8b | 51.55326 | 40.5584 | 46.60696 | 136.9404 | 3.376375 | 2.938196 |
|  | Prdx1 | 57.23657 | 188.8787 | 52.99063 | 554.0668 | 2.933453 | 10.45594 |
|  | Txnrd1 | 235.6753 | 530.74 | 314.95 | 1542.479 | 2.90628 | 4.897537 |
|  | Slc39a4 | 177.0612 | 201.8826 | 134.5994 | 577.145 | 2.858815 | 4.287872 |
|  | Rnf128 | 72.66212 | 101.318 | 70.04205 | 286.5112 | 2.827842 | 4.090559 |
|  | Gm614 | 33.89548 | 26.33004 | 48.46707 | 135.9928 | 5.164928 | 2.80588 |
|  | Smpdl3b | 63.08896 | 46.28781 | 67.60518 | 185.5852 | 4.009376 | 2.745133 |
|  | Mitf | 114.0486 | 154.4638 | 173.5946 | 475.3533 | 3.077441 | 2.738295 |
|  | Lipa | 549.0966 | 828.5328 | 1311.137 | 3539.431 | 4.271926 | 2.699512 |
|  | Tyk2 | 145.2759 | 173.8264 | 160.3693 | 465.9341 | 2.680457 | 2.905382 |
|  | Treml4 | 389.1219 | 264.9583 | 445.7987 | 1193.765 | 4.505482 | 2.677812 |
|  | Cdc42ep2 | 326.1657 | 317.357 | 345.3064 | 916.1659 | 2.886862 | 2.653197 |
|  | Fcgr2b | 196.4993 | 374.9906 | 317.8782 | 991.3807 | 2.643748 | 3.118744 |
|  | Ptgr1 | 57.76782 | 62.05118 | 57.55167 | 160.3673 | 2.584435 | 2.786492 |
|  | Ggct | 87.66655 | 81.34524 | 73.27348 | 205.8013 | 2.529973 | 2.808673 |
|  | Adss | 142.1381 | 159.7049 | 145.1067 | 401.0851 | 2.511413 | 2.76407 |
|  | Ube2f | 501.2782 | 507.4941 | 485.732 | 1267.287 | 2.497146 | 2.609025 |
|  | Lair1 | 176.0003 | 117.3715 | 298.0887 | 741.5017 | 6.317562 | 2.487521 |
|  | Tnip1 | 412.5275 | 544.222 | 521.4573 | 1343.443 | 2.468557 | 2.576324 |
|  | Blvrb | 220.8792 | 219.9991 | 209.2519 | 541.061 | 2.459378 | 2.585692 |
|  | Havcr2 | 30.88945 | 81.93967 | 51.45104 | 200.611 | 2.448276 | 3.899065 |
|  | Armcx3 | 170.2139 | 226.7431 | 202.0987 | 553.2256 | 2.439879 | 2.737403 |
|  | Fas | 475.6779 | 565.5691 | 707.1131 | 1703.505 | 3.012019 | 2.409098 |
|  | P4ha1 | 193.7425 | 551.579 | 564.0028 | 1351.688 | 2.450579 | 2.396598 |
|  | Acp2 | 153.4633 | 218.3806 | 180.8706 | 523.3156 | 2.396347 | 2.893314 |
|  | Nod1 | 353.6943 | 347.921 | 295.7029 | 831.0121 | 2.388508 | 2.810295 |
|  | Setd8 | 611.2473 | 614.2613 | 615.5673 | 1464.901 | 2.384817 | 2.379757 |
|  | Relb | 227.5722 | 406.4109 | 350.3289 | 961.7145 | 2.36636 | 2.745176 |
|  | Armcx3 | 126.2354 | 159.5058 | 156.6938 | 376.0707 | 2.357724 | 2.400035 |
|  | Ikbkg | 211.301 | 209.1945 | 258.031 | 604.5546 | 2.889916 | 2.342954 |
|  | Armcx3 | 136.3409 | 180.1906 | 156.5402 | 420.2921 | 2.332487 | 2.684882 |
|  | Nfkbia | 1413.464 | 2259.403 | 2197.371 | 5227.288 | 2.313571 | 2.378883 |
|  | Rnf19b | 691.0174 | 588.2897 | 890.457 | 2019.031 | 3.432035 | 2.267409 |
|  | Adam17 | 632.0676 | 1094.859 | 921.502 | 2478.497 | 2.263759 | 2.689627 |
|  | Usp40 | 100.7343 | 177.0386 | 165.2499 | 394.1694 | 2.226461 | 2.385292 |
|  | Traf3 | 270.7344 | 293.9367 | 283.3024 | 651.8859 | 2.217777 | 2.301025 |
|  | Acsl5 | 384.1707 | 378.9886 | 373.1854 | 836.153 | 2.206275 | 2.240584 |
|  | Esd | 199.3319 | 587.07 | 377.7107 | 1292.141 | 2.201 | 3.420981 |
|  | Mocos | 267.9274 | 198.332 | 300.5315 | 659.2131 | 3.323785 | 2.193491 |
|  | 4930402H24Rik | 161.1633 | 171.555 | 174.9606 | 381.3746 | 2.223046 | 2.179774 |
|  | Sms | 49.31472 | 56.83111 | 54.15987 | 123.7019 | 2.176658 | 2.284013 |
|  | Mllt6 | 135.6279 | 218.5371 | 150.3352 | 475.3457 | 2.175126 | 3.161906 |
|  | Pfkp | 362.4844 | 1019.796 | 1047.143 | 2265.61 | 2.221632 | 2.163611 |
|  | Gm14005 | 68.45878 | 68.47953 | 93.74505 | 200.4686 | 2.927423 | 2.138444 |
|  | 1700029I01Rik | 177.3064 | 199.0173 | 204.7527 | 437.4675 | 2.198137 | 2.136565 |
|  | Gm13251 | 288.2782 | 353.3414 | 283.3991 | 748.37 | 2.11798 | 2.640694 |
|  | Tubgcp4 | 134.3361 | 121.0239 | 168.1567 | 355.8614 | 2.940423 | 2.116249 |
|  | Ube2f | 191.0339 | 213.5145 | 205.139 | 445.4892 | 2.086459 | 2.171646 |
|  | Chka | 68.08365 | 117.8167 | 89.89751 | 244.0595 | 2.071518 | 2.714864 |
|  | Esd | 379.508 | 912.9429 | 617.9561 | 1878.006 | 2.057091 | 3.039061 |
|  | C3 | 1738.083 | 1624.211 | 2452.225 | 5031.343 | 3.097715 | 2.051746 |
|  | Pion | 1004.283 | 814.3842 | 1294.005 | 2639.729 | 3.24138 | 2.039968 |
|  | Nfkb2 | 419.0714 | 757.4872 | 733.0349 | 1540.429 | 2.033604 | 2.101441 |
|  | BC016495 | 91.09979 | 138.9999 | 209.5864 | 424.6094 | 3.054745 | 2.025939 |
|  | Traf1 | 52.71349 | 150.5218 | 64.8663 | 304.4527 | 2.022649 | 4.693542 |
|  | Pgm2 | 192.1379 | 361.6845 | 247.3269 | 727.0406 | 2.010151 | 2.939593 |
|  |  |  |  |  |  | UA/SF | UA/TG |
| UA | Mmp19 | 41.86348 | 142.8492 | 1146.016 | 139.4044 | 8.022558 | 8.220801 |
|  | Cysltr1 | 39.37074 | 64.95506 | 201.8095 | 57.33799 | 3.106909 | 3.519647 |
|  | Arhgap5 | 177.8474 | 115.4466 | 444.1297 | 144.9867 | 3.847058 | 3.063245 |
|  | A430084P05Rik | 68.21492 | 70.1137 | 207.081 | 76.70006 | 2.953502 | 2.69988 |
|  | Tctn1 | 207.39 | 133.1707 | 451.9974 | 174.5917 | 3.394121 | 2.588883 |
|  | Ly6a | 298.0309 | 312.4899 | 774.0067 | 254.9333 | 2.476901 | 3.036114 |
|  | Ltb4r1 | 188.9238 | 128.155 | 583.2604 | 241.6079 | 4.551212 | 2.414078 |
|  | Ccdc80 | 87.02098 | 74.57926 | 174.5461 | 58.68448 | 2.34041 | 2.974314 |
|  | D830046C22Rik | 63.00746 | 51.02611 | 238.0263 | 101.7684 | 4.664793 | 2.338902 |
|  | Naaa | 559.0213 | 552.4344 | 1385.656 | 624.8451 | 2.508273 | 2.2176 |
|  | Entpd3 | 308.9479 | 319.0412 | 702.8953 | 198.8127 | 2.203149 | 3.535465 |
|  | Siglec5 | 502.5297 | 564.4392 | 1232.756 | 533.6249 | 2.184036 | 2.310154 |
|  | Galnt6 | 119.2814 | 193.8484 | 406.8153 | 152.2815 | 2.098627 | 2.67147 |

All genes for which expression (first 4 columns of numbers) was at least 2-fold higher in one activating condition compared to both other activating conditions and to blood (BL) are shown, ordered by the minimum fold-difference between conditions (last 2 columns). SF = synovial fluid; TG = thioglycollate; UA = uric acid.
