# Supplementary material for: Gene Expression during the Generation and Activation of Mouse Neutrophils: Implication of Novel Functional and Regulatory Pathways
Source: PLoS One. 2014 Oct 3;9(10):e108553. doi: 10.1371/journal.pone.0108553 (PMC4184787; doi:10.1371/journal.pone.0108553)
Supplement: Text S1 — Comparison of neutrophils activated by different stimuli. (DOCX) [file pone.0108553.s004.docx]

**Text S1. Comparison of neutrophils activated by different stimuli**

To compare the three stimulated neutrophil populations (SF, TG, and UA) with circulating blood neutrophils, the dataset was limited to the 1283 genes differing across these 4 conditions (ANOVA P<0.01) with fold-change > 2 in at least one pair-wise comparison, and within-group CV<0.05. Gene expression patterns were similar between TG and UA (r=0.79) but greater in magnitude for TG as indicated by the slope of the regression line (**Fig. S2A**). Correlation was still significant but lower comparing TG and SF (r=0.71) and much lower comparing SF and UA (r=0.55)(**Fig. S2A**).

Venn diagrams at different fold-change cut-offs corroborated the assessment that changes in expression were greatest with TG and less similar comparing SF to UA than comparing TG to either UA or SF. Genes up-regulated at least 2-fold versus blood neutrophils were more numerous in TG (n=444) and SF (n=305) than UA (n=147) neutrophils, and there was much more overlap between TG and UA (n=110) or TG and SF (n=190) than SF and UA (n=55), and all but 3 genes in the latter group were also increased in TG (**Fig. S2B**). Relaxing criteria so that only one condition required fold change > 2, while the others were allowed fold change > 1.5, increased the numbers of shared genes as expected, but most prominently for genes shared by TG and UA or by all 3 categories, and the number of genes shared by SF and UA but not TG remained small (n=7)(**Fig. S2B**). Down-regulated genes were most numerous with SF (n=291), followed by TG (n=249), then UA (n=187). Use of relaxed criteria indicated that down-regulation of genes was more similar across all 3 conditions than was up-regulation, since 49% of genes were down-regulated in all 3 conditions, compared to 26% of up-regulated genes (**Fig. S2B**). Construction of Venn diagrams across a wide range of fold-change cut-offs gave analogous results (data not shown), ensuring that this interpretation was not an artifact of the arbitrary cut-off values chosen.

Similarities and differences between gene expression patterns in SF, TG, and UA neutrophils were also analyzed according to enrichment for particular functions in the Gene Ontology Biological Process database, using the analysis tool DAVID with significance defined as Q<0.05 after adjustment for multiple comparisons (Benjamini-Hochberg method), and with inclusion of a clustering step to identify redundant terms. Using lists of genes up-regulated 1.5- or 2-fold comparing SF, TG, or UA to blood neutrophils, 23 clusters of related terms were identified as having significant enrichment in at least one dataset. Representative terms from these 23 clusters were chosen for comparison of the proportions of genes found within them among genes up-regulated in SF, TG, and UA. Using data limited to fold-changes > 2, 5 terms showed no differences between groups, 17 were enriched in TG compared to UA, 8 in SF compared to UA, and 4 in TG compared to SF. However, the numbers of genes with fold-change > 2 were much higher in TG (n=303) than SF (n=222) or especially UA (n=99). When the analysis was repeated using the top 200 genes in each list (corresponding to fold changes of 2.44 for SF, 2.84 for TG, and 1.75 for UA), 21 GO terms in 6 clusters were significantly enriched in at least one condition, and although enrichment was nominally greater with TG (all 6 clusters having Q<0.05, compared to 2 in SF and 1 in UA), there was no significant difference when the groups were compared to each other. Thus, analysis of up-regulated genes based on function provided additional evidence that differences between the 3 activated neutrophil populations were largely quantitative rather than qualitative.

Lists of genes down-regulated in SF, TG, or UA relative to blood neutrophils were analyzed in the same way. Although down-regulated genes were nearly as numerous as up-regulated genes (see **Fig. S2B**), they were not distributed as clearly into functional groups. Only 3 terms, redundant and consisting of multiple genes for histones, showed significant enrichment in any condition, and enrichment did not differ significantly among SF, TG, and UA neutrophils.

Because analysis using DAVID was only sensitive for finding enrichment in relatively large groups of related genes, we also identified every gene with expression in TG, SF, or UA that was at least 2-fold higher than in both of the other conditions as well as in blood. Seventy-nine genes were relatively specific for TG, 49 for SF, and 13 for UA (see **Table S2**). Inspection of these lists revealed several groups of genes with shared functions. TG neutrophils up-regulated NFκB subunits and regulators, enzymes involved in gluthathione metabolism and other antioxidants, and signaling molecules in pathways for responding to microbial products. SF neutrophils up-regulated MHC class II genes, the C1q component of complement, all 3 members of the Nr4a nuclear hormone receptor subgroup (*Nr4a1*, *2*, and *3*), and molecules related to the uptake and metabolism of lipoproteins. UA neutrophils up-regulated two receptors for leukotrienes (*Cysltr1* and *Ltb4r1*). All of these enrichments were supported by analyses using DAVID and the GO and KEGG databases, with unadjusted P<0.05 for relevant categories/pathways but with adjusted Q>0.05 reflecting the small numbers of genes in each category/pathway and the large numbers of categories being tested. Genes specifically down-regulated in TG (n=3), SF (n=42), or UA (n=9) did not contain any shared functions that were obvious on inspection, nor by analysis using DAVID.
