# Supplementary figures and images for: Gene Expression during the Generation and Activation of Mouse Neutrophils: Implication of Novel Functional and Regulatory Pathways
Source: PLoS One. 2014 Oct 3;9(10):e108553. doi: 10.1371/journal.pone.0108553 (PMC4184787; doi:10.1371/journal.pone.0108553)

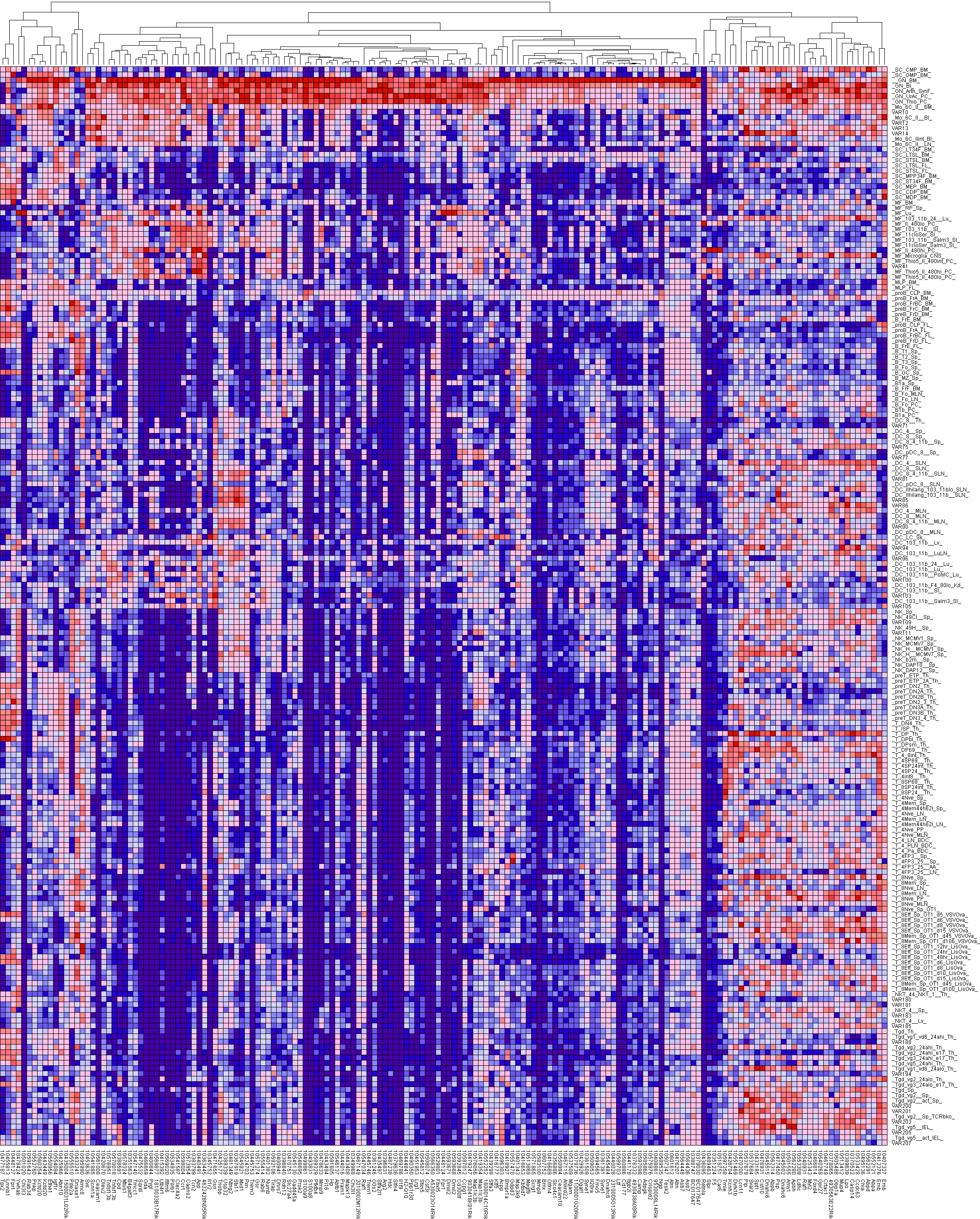

Supplement: File S1 — Expression, in purified leukocyte populations in the ImmGen database, of genes comprising a neutrophil signature in the BioGPS database [47]. All genes in the BioGPS signature for which there were comparable data in ImmGen are shown. Blue indicates relatively low expression, red indicates high expression; expression data were log-transformed and mean-centered for each gene (i.e., row-normalized) using the HeatMapImage module of GenePattern. Gene names are shown on the right, hierarchical clustering of the expression patterns on the left, and ImmGen populations (not clustered) along the top; text can be viewed using a photo viewer with a magnification function. Neutrophil-related populations at the left edge include common myeloid precursor (SC_CMP_BM), granulocyte-monocyte precursor (SC_GMP_BM), bone-marrow neutrophils (GN_BM), blood neutrophils (GN_Bl), neutrophils from inflamed synovial fluid (GN_Arth_SynF), uric-acid-induced peritoneal neutrophils (GN_UrAc_PC), and thioglycollate-induced peritoneal neutrophils (GN_Thio_PC). (PNG) [file pone.0108553.s005.png]
